# Decision aids to help older people make health decisions: a systematic review and meta-analysis

**DOI:** 10.1186/s12911-016-0281-8

**Published:** 2016-04-21

**Authors:** Julia C. M. van Weert, Barbara C. van Munster, Remco Sanders, René Spijker, Lotty Hooft, Jesse Jansen

**Affiliations:** Amsterdam School of Communication Research/ASCoR, Department of Communication Science, University of Amsterdam, P.O. Box 15791, 1001 NG, Amsterdam, The Netherlands; University Medical Center Groningen (UMCG), Department of Medicine, Groningen, The Netherlands; Gelre Hospitals, Department of Geriatrics, Apeldoorn, The Netherlands; Cochrane Netherlands, Julius Center for Health Sciences and Primary Care, University Medical Center Utrecht, Utrecht, The Netherlands; Medical Library, Academic Medical Centre, University of Amsterdam, Amsterdam, The Netherlands; Sydney School of Public Health, Centre for Medical Psychology and Evidence-based Decision-making (CeMPED), University of Sydney, Sydney, Australia

**Keywords:** Medical decision making, Shared decision making, Decision aid, Decision support tool age-differences, Gerontology, Communication, Health education, Informed choice

## Abstract

**Background:**

Decision aids have been overall successful in improving the quality of health decision making. However, it is unclear whether the impact of the results of using decision aids also apply to older people (aged 65+). We sought to systematically review randomized controlled trials (RCTs) and clinical controlled trials (CCTs) evaluating the efficacy of decision aids as compared to usual care or alternative intervention(s) for older adults facing treatment, screening or care decisions.

**Methods:**

A systematic search of (1) a Cochrane review of decision aids and (2) MEDLINE, Embase, PsycINFO, Cochrane library central registry of studies and Cinahl. We included published RCTs/CCTs of interventions designed to improve shared decision making (SDM) by older adults (aged 65+) and RCTs/CCTs that analysed the effect of the intervention in a subgroup with a mean age of 65+. Based on the International Patient Decision aid Standards (IPDAS), the primary outcomes were attributes of the decision and the decision process. Other behavioral, health, and health system effects were considered as secondary outcomes. If data could be pooled, a meta-analysis was conducted. Data for which meta-analysis was not possible were synthesized qualitatively.

**Results:**

The search strategy yielded 11,034 references. After abstract and full text screening, 22 papers were included. Decision aids performed better than control resp. usual care interventions by increasing knowledge and accurate risk perception in older people (decision attributes). With regard to decision process attributes, decision aids resulted in lower decisional conflict and more patient participation.

**Conclusions:**

This review shows promising results on the effectiveness of decision aids for older adults. Decision aids improve older adults’ knowledge, increase their risk perception, decrease decisional conflict and seem to enhance participation in SDM. It must however be noted that the body of literature on the effectiveness of decision aids for older adults is still in its infancy. Only one decision aid was specifically developed for older adults, and the mean age in most studies was between 65 and 70, indicating that the oldest-old were not included. Future research should expand on the design, application and evaluation of decision aids for older, more vulnerable adults.

**Electronic supplementary material:**

The online version of this article (doi:10.1186/s12911-016-0281-8) contains supplementary material, which is available to authorized users.

## Background

Medical decisions for older adults are often complex. The evidence base to support the use of many decision support interventions in older people is limited, especially for older people experiencing multi-morbidity, cognitive impairment or frailty as these groups have been systematically underrepresented in randomized controlled trials (RCTs) [1]. In addition, treatments frequently have adverse effects in older adults. The extent to which these effects may occur is difficult to predict in this heterogeneous population, in particular in people with multi-morbidity who are already on multiple medications, implying underreporting of harms [2]. Most health decisions for older people need to carefully view the evidence on potential harms and benefits in light of decreasing life expectancy. This often involves trading off quantity of life for quality of life. These types of decisions when there is limited evidence about outcomes, need to trade off benefits and harms and the decision-making process needs to be highly individualized are considered ‘preference sensitive’.

Making a decision, especially a preference sensitive one, requires certain skills. Simplified, decision makers first need to acquire information, then they have to identify, understand and evaluate the different options, and finally they need to use a suitable strategy to select the option with the best outcome. Several papers have outlined how age-related cognitive and affective changes may influence these skills [3–5]. Until now, theory-based research on improving decision-making in older populations is largely missing [3]. Empirical studies have shown that, compared with younger individuals, older adults tend to seek less information to make a decision and make decisions faster [4, 6]. Older adults also report preferences for fewer choice options [6], have greater difficulties in understanding information about available options [7] and tend to disproportionally focus on emotional aspects (often positive information) when making a decision [8]. Moreover, older peoples’ preferences will vary widely depending on for example their frailty, level of education, cognitive and health status [9]. A systematic review found that the majority of people, including older people, prefer involvement in medical decision-making [10], although a subset of patients, usually older and less educated, prefer to delegate decisions to their clinician [11].

This complex decision-making process can be supported by using decision aids. Decision aids are “*intended to help people participate in decisions that involve weighing the benefits and harms of treatment options often with scientific uncertainty”* [12, page 1]. However, existing decision aids, if theory-based [13, 14], rely on findings and evidence concerning younger patients (e.g., [15–17]). While these tools have been overall successful in enhancing the quality of the decision making process [12, 18], it is unclear whether the results also apply to older adults (i.e., adults aged 65+), hence whether decision aids are effective tools for this target group. Research is urgently needed to investigate whether older patients maximally benefit from decision aid-usage. The aim of the current study is therefore to find out whether the results from previous high quality studies among people of all ages [see 12 for a review] also hold for older people by conducting a systematic review of randomized controlled trials (RCTs) or clinical controlled trials (CCTs) evaluating the effectiveness of decision aids as compared to usual care and/or alternative intervention(s) for older adults facing treatment, screening or care decisions, either for themselves or for an incapacitated significant other, on attributes of the decision and the decision process. Secondary outcomes are behavioral, health, and health system effects.

## Method

### Criteria for considering studies for this review

The method used in this study was based on the Cochrane review “*Decision aids for people facing health treatment or screening decisions*” [19], from now on referred to as “the Cochrane review”, with modifications made on the type of participants and the type of interventions to suit our aims. We followed an internal protocol that was written at the start of the study.

#### Types of participants

We included studies that evaluated a decision aid in a sample with a mean age of 65 years or older OR reported effectiveness analysis of the decision aid in a subsample of participants aged 65+ years. We included decision aids for surrogates making a decision for an incapacitated significant other when the mean age of participating surrogates or the mean age of the incapacitated significant others was 65+ years. We included studies in which participants were making an actual decision or a hypothetical decision.

#### Types of interventions

Decision aids were defined as “*interventions designed to help people make specific and deliberative choices among options (including the status quo) by making the decision explicit and by providing (at the minimum) information on the options and outcomes relevant to a person’s health status*” [based on 19, page 4]. While the Cochrane review [19] only included studies that evaluated decision aids for people facing treatment or screening decisions, we also included studies on the efficacy of decision aids to help people make care decisions (e.g., advanced care planning). We excluded decision aid studies focusing on: decisions about lifestyle changes, clinical trial entry, general advance directives, general education programs; and decision aids to promote a recommended option.

#### Types of comparisons

We included studies in which the use of a detailed decision aid was compared with usual care, alternative interventions, or a combination (e.g., a detailed decision aid could be compared with usual care and with a simple decision aid).

#### Types of outcomes

We evaluated outcomes related to the International Patient Decision Aid Standards (IPDAS) criteria for evaluating the effectiveness of decision aids [20–23].

##### Primary outcomes

Based on the IPDAS criteria: 1) attributes of the choice made: e.g., knowledge, accurate risk perceptions and informed choice, i.e., an (intended) decision that is based on sufficient knowledge combined with consistent attitudes and intentions [24]; 2) attributes of the decision-making process: e.g., helping the person to recognize that a decision needs to be made, decisional conflict, patient-provider communication, participation in decision making, satisfaction.

##### Secondary outcomes

Choice, adherence to chosen option, preference linked health outcomes (i.e., anxiety, worry, caregiver burden, depression, self-efficacy, decisional regret) and other health (service) outcomes mentioned.

#### Types of study design

We included all published studies using a RCT or CCT design.

### Search methods for identification of studies

The search method included:

- Searching the Cochrane review [19] for studies published before December 2009 describing interventions specifically developed for older people OR studies describing analysis of the effectiveness of decision aids in subgroups of people aged 65+.
- Conducting a follow-up search from 2009 onwards in the following databases using the search strategy of the Cochrane review as developed by Stacey et al. [19] and retrieved from the first author: MEDLINE (OvidSP) (December 2009-February 2014), Embase (OvidSP) (December 2009-February 2014), PsycINFO (OvidSP) (December 2009-February 2014), Cochrane library central registry of studies (Whiley) (2010–2014) and Cinahl (EBSCO HOST) (1937-February 2014). See Additional file 1 for details of the sources searched, the search strategies used and the number of hits retrieved.
- Snowball method (reference checking).

### Data collection and analysis

All references were collated in Endnote and duplicate records removed. Deduplicated records were subsequently loaded into Eppi reviewer software [25] for screening and data extraction. The priority screening module in Eppi reviewer [26] was used to assist the screening on title and abstract for inclusion. In short, a random sample of records (the training set), was assessed on title and abstract by two researchers individually (AK and RS) and inconsistencies were discussed and resolved with three authors (JJ, BM, JW). The abstracts were labeled for inclusion or exclusion for the next stage. This training set was used for machine learning using a support vector machine (SVM) classifier to classify instances and a class membership probability score was used to compile a ranked list of the remaining records (see results for further explanation).

Subsequently, three authors (JJ, BM, JW) screened full text papers and extracted data independently. The extent to which the decision aids under investigation fulfilled IPDAS criteria [20–22] and the risk of bias (double coded for each included study) were also assessed, using the Cochrane tool for judging risk of bias [27] and the EPPI software [25]. Inconsistencies were resolved by discussion amongst three authors (JJ, BM, JW).

We conducted meta-analysis, if possible (which was the case for knowledge, risk perception, decisional conflict, participation in decision making and choice). Following the method used in the Cochrane reviews [12, 19], we pooled results across studies in cases where: a) comparable outcome measures were used; b) the effects were expected to be independent of the type of decision studied; and c) similar comparisons were used (e.g., decision aids to usual care or detailed decision aids to simple decision aids). To facilitate pooling of data for some outcomes (e.g., knowledge, decisional conflict), the scores were standardized to range from 0 to 100 points [12]. We used Review Manager 5.3.5 software (RevMan 2014) to estimate a weighted treatment effect (with 95 % confidence intervals). For continuous measures, mean differences (MD) were used; for dichotomous outcomes, pooled relative risks (RR) were calculated, using random-effects models. Additional file 3 shows the results of the meta-analysis. Data for which meta-analysis was not possible were synthesized qualitatively. This was done by carefully summarizing the reported effects on the various outcomes per study, comparing the effects per outcome, and drawing conclusions about the effectiveness. Doubts about the interpretation were resolved by discussion amongst four authors (LH, JJ, BM, JW).

## Results

**Fig. 1:**
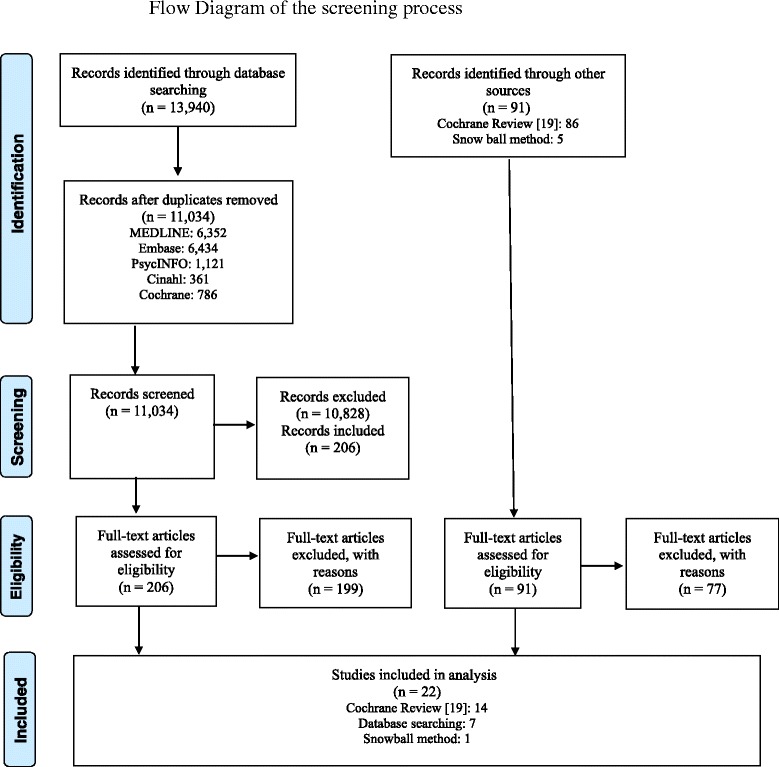
Provides the study flow diagram

Approximately 1 % of the total records identified through database searching in the Cochrane review [19] was assessed as potentially relevant for the current review on the efficacy of decision aids for older people after title and abstract screening. Using the 1 % frequency to calculate a sample size 1,207 records were randomly drawn from the 11,034 search results. Two reviewers independently screened the sample set against inclusion and exclusion criteria. In total, 25 records were identified as possibly relevant and 1,182 records were assessed as non-relevant. These results were subsequently used as a training set for the SVM classifier within Eppi reviewer to produce a ranked priority list drawn from the remaining 9,833 records. The ranked list was screened manually until the number of relevant records identified leveled off. When 1000 consecutive records did not result in any additional relevant citations screening was stopped. The 206 identified records was within the 99 % confidence interval based on extrapolation of the random sample outcome (calculated using nQuery advisor 7.0 software). We manually screened 55 % of the total number of search results which is on the safe upper boundary as described in Miwa et al. [26]. Taken together the 99 % CI and the high number of screened items it was deemed appropriate to stop screening the tail. In total, 5,969 out of 10,828 excluded abstracts were manually excluded and the remaining 4,859 were system excluded (Fig. 1).

The Cochrane review [19] included thirteen papers using a sample with a mean age of 65+ and one paper in which a subgroup analysis was conducted in a sample aged 65+ years. The additional search strategy yielded (after having removed 2,906 duplicates) 11,034 abstracts. After subsequent title and abstract screening, 10,828 were excluded and the full-text content of the remaining 206 papers was screened. Seven of those fulfilled all inclusion criteria. In addition, one paper was added using the snowball method. Hence, the search resulted in 22 citations that fulfilled the inclusion criteria [28–49]. In the meantime, an updated Cochrane review was published, for which databases were searched until June 2012 [12]. When comparing the included papers in our review (based on literature search until February 2014) with those included in the updated Cochrane review, we missed no papers that fulfilled our inclusion criteria. Three [32, 39, 46] of the eight [31, 32, 36, 39, 42, 45–47] papers that were yielded by our additional search were also included in the updated Cochrane review.

Additional file 2 gives an overview of the characteristics of the included papers. The 22 papers investigated the effectiveness of seventeen different decision aids using nineteen different samples. In six papers, the same three decision aids were evaluated [33 resp. 48; 34 resp. 44; 40 resp. 41]. The papers of Jones et al. [33] resp. Weymiller et al. [48] and Partin et al. [40] resp. Partin et al. [41] reported on exactly the same sample. Kaner et al. [34] used a subsample of Thomson et al. [44]. In three other papers [45–47], the same decision aid was studied, with the explanation that the two minute verbal video narrative [45, 46] was extended to six minutes in a later study [47]. The effectiveness was studied in three different samples [45–47].

Thirteen of the seventeen decision aids aimed to support a certain patient group or surrogates of patients in making a decision: four focused on patients with atrial fibrillation [31, 34, 35, 38, 44], two on patients with type II diabetes [33, 36, 48], one on patients with osteoporosis [39], one on breast cancer patients [43], one on prostate cancer patients [28] and one on people with self-reported knee pain [30]. The decision aid of Volandes et al. [45–47] faced elderly patients visiting a primary care or geriatric clinic with the possibility of advanced dementia, and the care choice they would like to make in that situation. In Volandes et al. [45] the concordance between patients and their surrogates for end-of-life preferences was investigated. In the other papers of Volandes et al. [46, 47] this only concerned the patients’ preferences. Two decision aids aimed to support carers or surrogates of people with dementia [32, 42]. Four decision aids were designed to support people in a screening decision: two were on colorectal cancer screening [29, 49], one on breast cancer screening [37] and one on prostate cancer screening [40, 41].

In nine studies the decision aid was delivered before a consultation [28–31, 35, 36, 40, 41, 43], in two studies during a consultation [34, 39, 44], in one study either before or during a consultation [33, 48], in five studies during a special research interview [32, 45–47, 49], and in three studies the decision aid was self-administered at home [37, 38, 42].

Eleven of the nineteen studies established a minimum age (‘cut-off point’) to be eligible for the study. In three studies participants had to be 50 years or older [29, 39–41], in two studies 60 years or older [30, 34, 44], in four studies 65 years or older [45–47, 49] and in one study they had to be 70 or 71 years old [37]. Hanson et al. [32] included surrogates of residents with advanced dementia who had to be 65 or older. In the other eight studies [28, 31, 33, 35, 36, 38, 42, 43, 48] there was no minimum age to be eligible for the study, although Fraenkel et al. [31] reported that the decision aid was developed for older patients.

In all studies, except for Street et al. [43] and Hanson et al. [32], the mean age of participants was 65 years or older. In Street et al. [43], using a sample with a mean age of 59.1, a comparison was made between older (65+), less educated patients, and younger (<65), more educated patients. In the study of Hanson et al. [32], the residents with dementia were on average 85.3 years, but the mean age of their surrogates was 59.0. In the study of Volandes et al. [45], both the elderly persons (mean age of 83) and their surrogates (mean age of 67.5) were on average older than 65. The study of Stirling et al. [42] also used a sample of carers with a mean age above 65 (i.e., 66.6), but did not mention the age of the patients with a diagnosis of dementia. Seven out of the remaining studies reported a mean age between 65 and 70 [28, 29, 33, 35, 36, 39–41, 48]; seven other studies used a sample with a mean age between 70 and 75 [30, 34, 37, 38, 44, 46, 47, 49]. Fraenkel et al. [31] did not report a mean age, but 56.3 % of the participants was aged 75 or older and 21.5 % was aged between 65 and 75.

### Attributes of the choice

Attributes of the choice was assessed by investigating whether the decision aid was effective with regard to knowledge, accuracy of risk perceptions and informed choice. Table 1 gives a summary of the findings.

**Table 1:** Summary of findings attributes of the choice: results in intervention group (IG) as compared with control group (CG) (*n* = 15)^a^

Short title	Knowledge	Risk perception	Informed choice
Fraenkel (2012) [31]	More knowledge of medications for reducing stroke risk.	More accurate estimates for risk of stroke and bleeding.	n.m.
	More knowledge of adverse effects (marginally significant).^b^		
Hanson (2011) [32]	More knowledge about dementia and feeding options.^b^	Fewer expected benefits from tube feeding.	n.m.
Jones (2009) [33]	More knowledge about statins and risk for coronary events, interacting with mode of delivery: Compared with the CG (pamphlet), patients whose clinicians delivered the decision aid during the office visit (IG2) showed significant more improvements in knowledge than when a researcher delivered the decision aid just before the office visit (IG1).	n.m.	n.m.
Man-Son-Hing (1999) [35]	More knowledge about stroke, atrial fibrillation, treatment and consequences.^b^	More correct quantitative estimates of stroke and bleeding risk when taking asparin or warfarin.^b^	n.m.
Mathers (2012) [36]	More knowledge about the treatment option that is most effective in reducing blood glucose level.^b^	More realistic expectations on the risk of hypoglycaemia, gaining weight and development of complications.^b^	
Mathieu (2007) [37]	More knowledge.	n.m.	A greater percentage of the IG women made an informed choice.
McAlister (2005) [38]	n.m.	More realistic estimates of the potential benefits and risks of warfarin and ASA (i.e. regarding biannual stroke risk in very-high-risk patients, RRR and biannual bleeding risk with warfarin and ASA).	n.m.
Montori (2011) [39]	More knowledge.^b^	More likely to correctly identify the 10-year fracture risk and to identify the estimated risk reduction with bisphosphonates.^b^	n.m.
Partin (2004) [40]	More knowledge in both IG1 (video) and IG2 (pamphlet) on prostate cancer natural history, treatment efficacy, and expert disagreement (the latter was higher in IG1 as compared to IG2).	n.m.	n.m.
	*No more knowledge on PSA accuracy.*		
Partin (2006) [41]	More prostate cancer screening knowledge in both IG1 (video) and IG2 (pamphlet).	n.m.	n.m.
Stirling (2012) [42]	More dementia knowledge according to authors (however *p* = .15, possibly due to small sample size).^b^	n.m.	n.m.
Thomson (2007) [44]	*Knowledge about warfarin improved in both the IG (decision aid) and CG (guidelines) post-clinic, but declined again in both groups by three months.*	n.m.	n.m.
	*No impact of either decision aid or guidelines on knowledge about aspirin.* ^b^		
Volandes (2009a) [45]	Knowledge scores increased for patients in both groups post intervention; however, the changes were higher in the IG (narrative plus video) than in the CG (narrative-alone).	n.m.	n.m.
	The change in knowledge scores was also higher for surrogates in the IG group.		
Weymiller (2007) [48]	*IG1 and IG2 (decision aid) and CG (pamphlet) scored similarly on knowledge.* Patients allocated to receive the interventions from their clinician during the visit (IG2) achieved better knowledge scores when using the decision aid than when using the control pamphlet (IG2); this effect was significantly greater than the effect of the decision aid vs the control pamphlet in patients allocated to receive the interventions from the researcher before the visit (IG1/CG1).	IG1 and IG2 (decision aid) were more likely to accurately estimate the potential absolute risk reduction afforded by statin use than CG (pamphlet). Patients allocated to receive the interventions from the clinicians during the visit (IG2) were most accurate when reporting the relevant cardiovascular risk without statins when using the decision aid than when using the pamphlet (IG2); this effect was significantly greater than the effect of the decision aid vs the control pamphlet in patients allocated to receive the interventions from the researchers (IG1/CG1).	n.m.
Wolf (2000) [49]	n.m.	IG was able to gauge more accurately the positive FOBT predictive value of screening for getting cancer than CG. There was no difference in correct response rates between IG1 and IG2. There were also significant differences between IG1, IG2 and CG in the perceived efficacy of screening in reducing CRC mortality. CG rated the efficacy of screening higher than IG1 (relative risk reduction information), who rated it higher than IG2 (absolute risk reduction information).^b^	n.m.

n.m. = not measured; IG = intervention group; CG = control group

^a^Unless otherwise stated are the described results effects in the intervention group (IG) as compared to the control group (CG); see Additional file 2 for description of the CG intervention. Standard font indicates positive results (*p* < .05 unless otherwise stated) in favour of the IG; italic font indicates no significant results

^b^Included in meta-analysis

#### Knowledge

Thirteen papers (eleven studies) reported the effectiveness of the intervention on knowledge. Seven studies could be included in the meta-analysis (see Additional file 3 and Table 1). Five studies compared decision aids to usual care using a continuous knowledge measurement. Results showed that people exposed to a decision aid had higher average knowledge scores (MD 6.50; 95 % CI 0.76 to 12.25). Two studies compared decision aids to usual care using dichotomous knowledge measurements. People using the decision aid also had higher average knowledge scores (decision aid versus usual care pooled RR 1.71; 95 % CI 1.33 to 2.18). Four additional studies, described in six papers, could not be included in the pooled outcome [33, 37, 40, 41, 45, 48]. In two of these papers, coming from the same study [33, 48], the decision aid increased knowledge when the decision aid was delivered by the patients’ clinician during the visit. This effect was significantly greater than the effect in patients who received the decision aid from the researcher before the consultation, and also greater than a control pamphlet (either delivered by the researcher before the consultation or by the clinician during the consultation). Partin et al. [40] found a positive effect on knowledge about prostate cancer natural history, treatment efficacy and expert disagreement, but not about PSA accuracy. In the other three papers, only positive effects of using the decision aid on knowledge were reported [37, 41, 45].

##### Risk perception

Eight studies assessed the effectiveness of the intervention on risk perception. Four studies could be included in the meta-analysis (see Additional file 3 and Table 1). People who received a patient decision aid were more likely to have accurate risk perceptions than those who received usual care. The pooled relative risk (RR) of having accurate risk perceptions was 2.27 (95 % CI 1.27 to 4.06). The other four studies reported results that could not be pooled [31, 32, 38, 48]. They all found positive effects on risk perception in the intervention group as compared to the control group [31, 32, 38, 48]. In the study of Weymiller et al. [48], patients allocated to receive the decision aid from the clinician during the visit were most accurate when reporting the estimated 10-year cardiovasculair risk. This effect was significantly greater than when the interventions (experimental or control) were delivered by the researcher before the consultation.

##### Informed choice

One study measured informed choice as an outcome. In this study, women were classified as making an informed choice if they had adequate knowledge and clear values and expressed an intention to either continue or stop mammography screening. The percentage of women who were able to make an informed decision to either continue or stop screening was 73 % in the intervention group compared with 49 % in the control group, which was a significant difference [37].

### Attributes of the decision process

Findings regarding attributes of the decision process mainly concerned decisional conflict, patient-provider communication, participation in decision making and satisfaction (see Table 2 for a summary).

**Table 2:** Summary of findings attributes of the decision process: results in intervention group (IG) as compared with control group (CG) (*n* = 17)^a^

Short title	Decisional conflict^c^	Patient-provider communication	Participation in decision making	Satisfaction	Other process outcomes
Davison (1997) [28]	n.m.	n.m.	More active role in treatment decision making (assumed by participants)^b^	n.m.	n.m.
Dolan (2002) [29]	Less decisional conflict (total)^b^ In particular: • *No difference in uncertainty* ^b^ • Better informed^b^ • Better clarity of values^b^ • More effective decision making^b^ • *No difference in support* ^b^	n.m.	Increase in SDM (vs no increase in SDM in CG). Majority of patients who preferred a SDM process felt that the actual SDM process was consistent with this preference (vs half of the CG patients)^b^	n.m.	n.m.
Fraenkel (2007) [30]	n.m.	n.m.	Greater decisional self-efficacy (i.e. self-confidence in abilities to participate in SDM). Greater preparedness to participate in SDM. *Note*: older adults (≥75) may be among the most likely to benefit.	n.m.	n.m.
Fraenkel (2012) [31]	• Better informed^b^ • Better clarity of values^b^	More frequent discussion of risk of stroke and risk of major bleeding.	n.m.	n.m.	n.m.
Hanson (2011) [32]	Less decisional conflict for surrogates (total)^b^ In particular: • Less uncertainty^b^ • More effective decision making^b^ • Better score on “factors contributing to uncertainty”^b^	Increased communication about feeding options with providers (i.e. more feeding discussions with physician, nurse practitioner or physician’s assistant). *No differences in discussions with other nursing home staff.*	*Aa higher proportion felt involved in feeding decisions (83 % vs 77 %) but the difference was not significant.*	*No differences in satisfaction with decision-making*	n.m.
Jones (2009) [33]	*Less decisional conflict (total) when the decision aid was delivered during the visit by the clinician (IG2), but this difference was not significant.*	n.m.	n.m.	n.m.	n.m.
Kaner (2007) [34]	n.m.	*Duration: Computer-based decision aids (IG1 and IG2) significantly prolonged the consultations.* Non-verbal behavior: More nodding, smiling and tool-directed gaze in both IGs, and less head-shaking and pointing to patients.	n.m.	n.m.	n.m.
Man-Son-Hing (1999) [35]	*No differences in decisional conflict (total)* ^b^ In particular: • *No difference in uncertainty* ^b^ • Better informed^b^ • *No difference in clarity of values* ^b^ • *No difference in effective decision making* ^b^ • *No difference in support* ^b^	n.m.	*No differences in participation in decision making* ^b^	*No differences in satisfaction with the decision-making process.*	n.m.
Mathers (2012) [36]	Less decisional conflict (total)^b^ In particular: • Less uncertainty^b^ • Better informed^b^ • Better clarity of values^b^ • More effective decision making^b^ • *No difference in support* ^b^	n.m.	More autonomy in decision-making about treatment (IG patient was 1.23 times more likely to make an autonomous decision than CG patient). A smaller proportion in the IG described their decision as ‘passive’ or ‘collaborative’.^b^	n.m.	n.m.
Mathieu (2007) [37]	*No differences in total decisional conflict* ^b^ In particular: • *No difference in uncertainty* ^b^ • Better informed^b^ • Better clarity of values^b^ • *No difference in effective decision making* ^b^ • *No difference in support* ^b^	n.m.	n.m.	n.m.	*No differences in attitudes towards screening.*
McAlister (2005) [38]	Less decisional conflict (total)^b^ In particular: • Less uncertainty^b^ • Better informed^b^ • Better clarity of values^b^ • More effective decision making (trend *p* = .09)^b^ • *No difference in support* ^b^	n.m.	n.m.	n.m.	n.m.
Montori (2011) [39]	*No differences in decisional conflict (total)* ^b^	n.m.	Observed patient involvement in SDM was approximately double in IG than in CG.	*No differences in satisfaction with knowledge transfer (according to patients).* Greater satisfaction with knowledge transfer in the IG group, particularly ‘helpfulness of the information’, ‘would want other decisions’, ‘recommend to others’ (according to clinicians). Improved quality of the decision making process, particularly ‘patients’ informed choice’, ‘provider expects patients to stick with the decision’ and ‘provider believes patient is satisfied with the decision’ (according to clinicians).	*No differences in trust in clinician (according to patients).*
Partin (2004) [40]	n.m.	IG2 (pamphlet) subjects were more likely than controls to discuss screening with their provider, *but IG1 (video) subjects were not.*	n.m.	n.m.	n.m.
Stirling (2012) [42]	Less decisional conflict (total) according to authors (however not statistically significant, possibly due to small sample size)^b^ In particular: • *No difference in uncertainty.* • *No difference in feeling informed.* • *No difference in clarity of values.* • *No difference in effective decision making.* • *No difference in support.*	n.m.	n.m.	n.m.	n.m.
Street (1995) [43]	n.m.	*No difference between IG and CG on patient-provider communication, and no interaction between age/education and intervention. In both groups, college-educated patients younger than 65 years of age were more active participants in the consultations than were older, less educated patients (*i.e. *asked questions more frequently, offered opinions, and expressed concerns).*	*No difference in involvement in decision making (self-reported) and no interaction between age/education and intervention.*	n.m.	n.m.
Thomson (2007) [44]	Less decisional conflict (total) immediately after the clinic. In particular: • *No difference in uncertainty.* • Better informed. • Better clarity of values. • *No difference in effective decision making.* • *No difference in support.* *No difference in decisional conflict subscales at three months.*	n.m.	n.m.	n.m.	n.m.
Weymiller (2007) [48]	*No significant differences in postvisit decisional conflict (total).* In particular: • *No difference in uncertainty.* • Better informed, particularly when the clinician delivered the intervention during the visit (IG2). • *No difference in clarity of values.* • More effective decision making. • *No difference in support.* *At 3 months, participants in the IG continued to have less decisional conflict than the IG, but these differences were no longer statistically significant.*	n.m.	n.m.	n.m.	n.m.

n.m. = not measured; IG = intervention group; CG = control group; SDM = shared decision making

^a^Unless otherwise stated are the described results effects in the intervention group (IG) as compared to the control group (CG); see Additional file 2 for description of the CG intervention. Standard font indicates positive results (*p* < .05 unless otherwise stated) in favour of the IG; italic font indicates no significant results

^b^Included in meta-analysis

^c^Decisional Conflict scale has five subscales and the possibility to calculate a total score; the table only includes results from subscales resp. a total score if reported in the paper

#### Decisional conflict

Twelve papers (eleven studies) reported findings on decisional conflict, using the Decisional Conflict Scale (DCS). This scale exists of five subscales, i.e., ‘feeling uninformed’, ‘ineffective decision making’, ‘feeling unclear about values’, ‘uncertainty’ and ‘feeling unsupported’. A total score measures the construct of overall decisional conflict [50]. A negative score indicates a decrease in decisional conflict, which is in favour of the IG using the decision aid.

In eleven papers, the total score of the DCS was calculated. In the eight studies that compared decision aids to usual care and could be pooled, the overall MD was −3.17 out of 100 points (95 % CI −4.44 to 1.90) (see Additional file 3 and Table 2). Of the three additional studies that could not be pooled, one reported a significantly positive effect [44]. The two others found *no* significant findings on total decisional conflict [33, 48], although Jones et al. [33] reported that there was less decisional conflict when the decision aid was delivered during the visit by the clinician, but this difference did not reach significance.

Nine studies reported on the effectiveness of decision aids on ‘feeling uninformed’ about options, benefits, and harms. When the decision aid was compared to usual care (*n* = 6), people exposed to the decision aid felt less uninformed (MD −4.88, 95 % CI −6.82 to–2.94) (see Additional file 3 and Table 2). In two of the three studies that could not be pooled, people exposed to the decision aid felt more informed than the CG [44, 48], but there was no difference in the third study [42]. Weymiller et al. [48], using the same sample as Jones et al. [33], but reporting on subscales, found that the intervention group felt particularly better informed when the clinician delivered the decision aid during the visit. This difference didn’t reach significance anymore after three months.

The ‘ineffective decision making’ subscale of the DCS was used in the same nine studies. Again, the data of six studies comparing decision aids with usual care could be pooled (see Additional file 3 and Table 2). Results showed a MD of −3.12 (95 % CI −5.03 to −1.21) in favour of the decision aid. In two out of the three additional studies, no significant differences were found between the IG and the CG [42, 44]. In line with the results on ‘feeling uninformed’, Weymiller et al. [48] reported that the intervention group particularly improved on ‘effective decision making’ when the clinician delivered the decision aid during the visit.

The ‘feeling unclear about values’ subscale of the DCS was also reported in nine studies, six of which compared decision aids to usual care and could be pooled (MD −3.01; 95 % CI −8.50 to 2.48) (see Additional file 3 and Table 2). Those exposed to the decision aid in the three studies that could not be pooled felt more clear about their values in one study [44], but not in the other two studies [42, 48].

The MD for ‘uncertainty’ was −3.65 (95 % CI −9.54 to 2.23) in six studies that compared decision aids to usual care (see Additional file 3 and Table 2). The three studies that could not be pooled reported no difference [42, 44, 48].

The ‘feeling unsupported’ subscale was measured in eight studies. In none of these there was a significant effect. MD was −1.30 (95 % CI −2.78 to 0.19) in five studies comparing decision aids to usual care (see Additional file 3 and Table 2). There was no difference in the three additional studies that could not be pooled [42, 44, 48].

#### Patient-provider communication

Five studies reported whether the intervention had an effect on patient-provider communication. In one study, the decision aid resulted in more frequent discussion about risk of stroke and major bleeding [31]. Three studies described mixed results, i.e., more communication about feeding options with physician, nurse practitioner or physicians’ assistant, but not with other nursing home staff [32], increased discussion of screening when using the pamphlet decision aid (IG2), but not when using the video decision aid (IG1) [40], positive effects on non-verbal communication, but not on verbal communication, and a prolongation of consultation duration [34]. The fifth study did not find positive effects of using a decision aid on patient-provider communication in older (65+) with lower levels of education. This study was the only study that looked at factors associated with involvement in communication and reported that younger (aged <65 years), higher educated patients were more active participants in the consultations (i.e., asked more questions, offered more opinions, produced more expressions of concern and had more active communication) than older, less educated patients, although the older, less educated patients did not perceive themselves as being less involved [43].

#### Participation in decision making

Eight studies described whether exposure to the decision aid had effects on participation in decision making: of these, four studies that compared the effects of decision aids to usual care using the Control Preferences Scale [51] could be pooled (see Additional file 3 and Table 2). The Control Preference Scale measures the role that the patient and the physician can assume in decision making, ranging from the patient selecting its own treatment through a collaborative model to a scenario where the physician alone makes the decision. The scale contains five response statements: two describe an active (patient-controlled) role, one a shared (collaborative) role, and two a passive (practitioner-controlled) role. For patients adopting an active (patient-controlled) role in decision making, the pooled RR for the four studies comparing decision aids to usual care was 1.30 (95 % CI 0.86 to 1.95) (see Additional file 3 and Table 2). For patients reporting a collaborative (shared decision making) role, there was no difference between decision aid and usual care (pooled RR 0.96; 95 % CI 0.82 to 1.13) (see Additional file 3 and Table 2). An increase in patient participation in decision making can also be reflected by a decrease in passive (practitioner-controlled) decision making. When comparing the decision aid versus usual care, a pooled RR of 0.61 (95 % CI 0.53 to 0.81) was found (see Additional file 3 and Table 2). Two out of the four additional studies that could not be pooled reported positive effects, i.e., greater decisional self-efficacy and preparedness to participate in decision making [30] and significantly more intense patient involvement in decision making according to video observations [39]. In the study of Hanson et al. [32], the intervention group surrogates felt more involved in feeding decisions than the control group (83 % vs 77 %), but this difference did not reach significance. In Street et al.’s study [43], patients’ perceptions of control over decision-making did not differ between groups or for patients differing in age and education.

#### Satisfaction

In four studies, satisfaction with decision making was assessed. In one study, ratings of the quality of the decisions were higher in the intervention group [29]. In three studies, there were no differences in satisfaction with the decision making process [32, 35] or with the knowledge transfer process (i.e., amount of information, clarity of information, helpfulness of information, would want other decisions, recommend-others) according to patients [39]. However, in the latter study, clinicians expressed greater satisfaction with the knowledge transfer process and with the quality of the decision making process [39].

#### Other outcomes

Mathieu et al. [37] found no differences in attitudes towards screening and Montori et al. [39] no differences in trust in the clinician.

### Behavior and health outcomes

The behavior and health outcomes that were reported in the included studies were categorized in choice, adherence with chosen option, preference-linked health outcomes, health outcomes and health services outcomes. Table 3 summarizes the results.

**Table 3:** Summary of findings behaviour and health outcomes: results in intervention group (IG) as compared with control group (CG) (*n* = 17)^a^

Short title	Choice	Adherence with chosen option	Preference-linked health outcomes (e.g. anxiety, depression, regret)	Health outcomes	Health services outcomes
Davison (1997) [28]	n.m.	n.m.	Lower state anxiety levels at 6 weeks. *No differences in levels of depression at 6 weeks.*	n.m.	n.m.
Dolan (2002) [29]	*No differences in decision (*i.e. *the proportion of colorectal cancer screening plans carried out)* ^b^	n.m.	n.m.	n.m.	n.m.
Fraenkel (2007) [30]	n.m.	n.m.	Greater arthritis self-efficacy.	n.m.	n.m.
Fraenkel (2012) [31]	A small proportion of the IG (*n* = 5) expressed a preference for medication that was not concordant with their current treatment plan. *No change in treatment plan.*	n.m.	*No differences in anxiety.* *No differences in worry about stroke and about bleeding.*	n.m.	n.m.
Hanson (2011) [32]	n.m.	After 3 months: Residents in the IG had greater use of some assisted oral feeding techniques (i.e. were more likely to receive a dysphagia diet and showed a trend toward greater staff eating assistance). *No differences in tube feeding after 9 months.*	*No differences in decisional regret for surrogates.*	Less weight loss after 9 months. *No differences in mortality.*	n.m.
Man-Son-Hing (1999) [35]	More IG patients made a definite choice about antithrombotic therapy (aspirin or warfarin). Slightly more IG patients preferred to continue taking aspirin rather than switch to warfarin. *A similar % of IG and CG reported that they, rather than their physician, made the decision.*	*After 6 months: A similar % of IG and CG continued to take the therapy that was initially chosen.*	n.m.	n.m.	n.m.
Mathers (2012) [36]	*No sign difference in proportion undecided (although patients in IG were three times more likely to change from undecided to decided).*	n.m.	n.m.	*No significant difference in the glycaemic control.*	n.m.
Mathieu (2007) [37]	IG women were less likely to be undecided. *Among those women who made a decision, there were no differences in intention to stop or continue screening.* *No differences in participation in screening.*	n.m.	*No differences in anxiety.* *No differences in breast cancer worry.*	n.m.	n.m.
McAlister (2005) [38]	After 3 months: Increase in the proportion of patients receiving therapy appropriate to their stroke risk (i.e. 12 % absolute improvement in IG as compared to CG).	*After 12 months: No difference in proportion of patients taking appropriate therapy (*i.e. *care in both IG and CG had regressed towards baseline levels).*	n.m.	n.m.	n.m.
Montori (2011) [39]	*No differences in distribution of prescriptions (bisphosphonates were started by 44 % of IG patients and 40 % of CG patients).*	*Adherence at 6 months was similarly high across both groups (self-reported), but the proportion with more than 80 % of days covered was higher in the IG (pharmacy records).*	n.m.	n.m.	n.m.
Partin (2004) [40]	IG1 and IG2 were less likely to intend to have a PSA. *2 weeks post-target appointment: No differences in PSA testing rates.*	*1 year post-target appointment: No differences in PSA testing rates.*	n.m.	n.m.	n.m.
Stirling (2012) [42]	n.m.	n.m.	*Less increase in carer burden (however not statistically significant, possibly due to small sample size).*	n.m.	n.m.
Thomson (2007) [44]	Participants in the IG not already on warfarin were much less likely to start warfarin than participants not already on warfarin in the CG.	n.m.	*There was a significant fall in anxiety immediately after the clinic, but no differences in reduction between IG and CG.*	*No differences in strokes and bleeds requiring admission.*	*No differences in GP consultations.* *No differences in hospital appointments.*
Volandes (2009b) [46]	IG group was more likely to prefer comfort care as their goal of care.	6 weeks after the intervention: IG had more stable preferences over time.			
Volandes (2011) [47]	IG group was more likely to prefer comfort care as their goal of care.	n.m.	n.m.	n.m.	n.m.
Weymiller (2007) [48]	30 % of IG patients and 21 % of CG patients not receiving statin at baseline started statin therapy immediately after the visit (not reported whether this difference was significant). IG patients with 10-year cardiovascular risk greater than 15 % most often started statin therapy.	Using the decision aid was not associated with stopping statin therapy and was associated with greater statin adherence at 3 months. Of 33 IG patients taking statin drugs at 3 months, 2 reported missing 1 dose or more in the last week compared with 6 of 29 patients in the CG group taking statin drugs. *Overall, there was no difference in adherence to patient choice at 3 months.*	n.m.	n.m.	n.m.
Wolf (2000) [49]	*No difference in screening interest between the two IGs and the CG* ^b^	n.m.	n.m.	n.m.	n.m.

n.m. = not measured; IG = intervention group; CG = control group

^a^Unless otherwise stated are the described results effects in the intervention group (IG) as compared to the control group (CG); see Additional file 2 for description of the CG intervention. Standard font indicates positive results (*p* < .05 unless otherwise stated) in favour of the IG; italic font indicates no significant results

^b^Included in meta-analysis

#### Choice or preference

Choice respectively preference was defined as the actual choice implemented respectively the participants’ preferred option. Whether or not the actual choice implemented is a ‘good’ choice can be difficult to determine. IPDAS reached agreement on criteria to judge “*the things that you would need to observe in order to say that after using a patient decision aid, the way the decision was made was good and that the choice that was made was good”* [12, page 7, based on 19–23]. This means that the chosen option can be considered appropriate if the choice matches with what the informed patient finds most important, and the decision aid can be considered effective when decision aid usage improved this match [12].

Fourteen studies reported whether exposure to the decision aid resulted in differences in choice as compared to the control group resp. usual care group. Only the data for choice for colorectal cancer screening could be pooled (see Additional file 3 and Table 3). Both two studies on colorectal cancer screening reported no difference between the IG and usual care. The pooled RR was 0.67 (95 % CI 0.18 to 2.54). Of the additional twelve studies that could not be pooled, six only reported effects in favor of the intervention group [38, 44–48]. In four studies, mixed results were described. Fraenkel et al. [31] found no changes in actual treatment plan. Although a small proportion of the intervention group expressed a preference for medication (aspirin) that was not in concordance with their current treatment plan (warfarin), audiotapes of the consultations revealed that these patients were convinced by their treating clinician to continue taking warfarin. Man-Son-Hing et al. [35] found no differences between the proportion participants in the intervention group and the control group that felt they, rather than the physician, made the decision, but more intervention group patients than control group patients made a definite choice about antithrombotic therapy (asparin or warfarin). As there was no right or wrong choice of antithrombotic therapy, the finding that the decision aid enhanced the patients’ ability to make choices regarding antithrombotic therapy was considered as a positive effect. Although Mathieu et al. [37] found that women in the intervention group were less likely to be undecided than women in the control group, there were no differences in intention to stop or continue screening and in participation in screening among the women that made a decision. Partin et al. [40] revealed that both intervention group participants were less likely to intend to have prostate cancer screening than the control group participants. However, there were no differences in actual PSA testing rates. In the other two studies [36, 39] there were no significant differences between the intervention and the control groups in the decisions made. Mathers et al. [36] noted that patients in the intervention group were over three times more likely to change from undecided to decided than in the control group, but this difference didn’t reach significance.

#### Adherence with chosen option

Seven studies measured adherence with chosen option. One reported only positive effects [46]. Intervention group participants were more likely to opt for comfort care as their preferred goal of care and this preference was more stable over time as compared with the control group. Two studies had mixed findings. Hanson et al. [32] found that, after three months, more assisted oral feeding techniques were used in the intervention group as compared with the control group, but after 9 months, there were no longer any differences. In the study of Weymiller et al. [48], there was no difference in adherence to choice at three months. However, of 33 patients in the intervention group taking statins at three months, two reported missing one dose or more in the past week, as compared to 6 out of 29 patients in the control group, indicating higher medication adherence in the intervention group. The other four studies found no effects of the intervention after six months [35, 39] or one year [38, 40].

#### Preference-linked health outcomes

In seven studies, preference-linked health outcomes, mainly anxiety or worry, were assessed. One study found a positive effect of using the decision aid on anxiety [28], but three other studies did not found effectiveness on anxiety or worry [31, 37, 44]. In one study, using the decision aid was effective in increasing arthritis self-efficacy [30]. In another study the intervention group of surrogates has less increase in caregiver burden than the control group [42], but this effect was not significant. No effectiveness was established on depression [28] or decisional regret [32].

#### Health outcomes

Three studies examined the effectiveness of using the decision aid on health outcomes. In the study of Hanson et al. [32], residents with dementia had less weight loss after nine months, but there were no differences in mortality. The other two studies found no effects on glycaemic control [36] or strokes and bleeds [44].

#### Health services outcomes

Only one study investigated health services outcomes. There were no differences in General Practitioner consultations and hospital appointments [44].

### IPDAS criteria

Additional file 4 shows the extent to which studies addressed issues considered important by International Patient Decision aid Standards (IPDAS) [21, 22].

All decision aids evaluated in the included studies fulfilled at least six IPDAS criteria. Eight IPDAS criteria (i.e., IPDAS 1, 2, 3, 6, 7, 9, 10 and 25) were fulfilled in 90 % or more of the decision aids. All decision aids (100 %) described the condition (health or other) related to the decision (IPDAS-1) and the decision that needed to be considered (IPDAS-2), provided information about the procedures involved (IPDAS-5), and, if dealing with screening, information about what the test was designed to measure (IPDAS-9) and possible next steps based on the test results (IPDAS-10). Four criteria were fulfilled in less than 25 % of the decision aids, i.e., information about detection and treatment of disease that would never have caused problems if screening had not been done (IPDAS-12), the date when the decision aid was last updated (IPDAS-26), whether authors of the decision aid or their affiliations stand to gain or lose by choices people make after using the decision aid (IPDAS-27) and reporting of readability levels (IPDAS-28). Two important criteria, i.e., whether value clarification was included (IPDAS-17) and whether the decision aid made it possible to compare the positive and negative features of the available options (IPDAS-18) were fulfilled in 68 % resp. 77 % of the decision aids. Of the thirteen decision aids in which probabilities were graphical presented, seven used the same scale in each graph; in five decision aids this was unclear (IPDAS-16).

In five decision aids [28, 30, 36, 38, 43], between 25 % and 50 % of the criteria were fulfilled. The other decision aids fulfilled 50 % or more of the criteria, five (described in seven papers) even more than 75 % [31, 33, 34, 37, 39, 44, 48]. No relationship could be found between the fulfillment of the IPDAS criteria and the effectiveness of the decision aid.

### Risk of bias

Additional file 5 gives an overview of the risk of bias for each individual paper, and the total risk of bias of included studies. There was variability in potential risk of bias across studies. The two criteria that were most often problematic were lack of blinding of participants and personnel (50 % no blinding and 32 % unclear) and the potential for selective outcome reporting (86 % unclear). Random sequence generation and providing complete data was without risk of bias in the majority (77 %) of the studies.

When looking at the individual papers, five had high risk of bias, based on over five out of seven items being unclear or judged with high risk [30, 36, 42, 43, 49]. In one paper [44] there were no indications at all of risk of bias. No relationship could be found between the risk of bias and the effectiveness of the decision aid.

## Discussion

This review shows promising results on the effectiveness of decision aids for older adults. Decision aids have the potential to increase older adults’ risk perception, improve knowledge, decrease decisional conflict, and improve patient participation in decision making by decreasing practitioner-controlled decision making. Regarding decisional conflict, particularly feelings of being informed, clarity of values and effective decision making seemed to improve.

These findings on knowledge, risk perception, decisional conflict and participation in decision making are in line with the results of the Cochrane review on the effectiveness of using a decision aids in a general population [12], i.e., people of all ages. Hence, the results of the Cochrane review seem to hold for older adults, although the effects on knowledge and decisional conflict, especially regarding the subscales ‘feeling uninformed’ and ‘ineffective decision making’, are less strong than in the Cochrane review (based on a comparison of the confidence intervals; see Additional file 3). This indicates that decision aids might be valuable tools for an older population and suggests that decision aids have potential to support older people’s information processing and decision making. However, there are a couple of concerns that can be raised from the results. First, this review shows that the body of literature on the effectiveness of decision aids for older adults is still in its infancy. Next to the relatively small number of papers that could be included in this review and the heterogeneity of the studies, only one paper explicitly stated that the decision aid was developed for older adults [31]. In seven studies (reported in eight papers), the mean age of the participants was between 70 and 75 [30, 34, 37, 38, 44, 46, 47, 49], and only one study reported that more than half of the participants was older than 75 [31]. In the other studies, the mean age was below 70, which indicates that the oldest-old (80+ year) were not included. Even though eleven of the nineteen studies established a minimum age (ranging from 50 to 70 years old) to be eligible for the study, it was not clear for most of the decision aids whether or how the decision aid was adapted to older adults’ abilities, needs or preferences. Incorporating theory into the development, design and evaluation of tools specifically designed to support decision-making, may potentially lead to better decision quality and outcomes [52]. This might especially be important in research on older adults, as this group is even more heterogeneous than the general population. However, hardly any attention was paid to whether or not the way of presenting choices was appropriate for older adults. Older adults are expected to have more difficulties in deliberative information processing, especially of factual and statistical information, which might result in miscomprehensions [4]. Their decision making processes may limit the amount and type of information they use and reduce their preferences for choice which has implications for the likelihood that they will make a truly informed decision. A person’s ability to use decision aids and participate in the decision making process will not only be determined by their skills, but also by the quality and suitability of the decision aids they have access to [53]. If designed age appropriately, decision aids could help compensate for these difficulties. As the evidence is inconclusive about how best to present information to older adults [see for example 54–58], newly developed decision aids should be carefully tested in the target group of older people. Moreover, according to life-span developmental literature, older adults’ motivation to put efforts in decisions depends on the perceived personal relevance of the decision and the person’s perceived self-efficacy. Improving perceived relevance and building self-efficacy might therefore be crucial elements of medical decision making interventions for older adults [59].

Only few of the included studies in the review conducted subgroup analysis in adults with low health literacy or numeracy, low educated adults, frail patients or other vulnerable (sub)groups. We therefore do not know whether the decision aids under investigation were also effective in these subgroups of older adults. In a recent patient-level meta-analysis of seven randomized trials, patients using a decision aid gained knowledge, were more likely to know their risk, and had less decisional conflict along with greater involvement in SDM compared with usual care. These gains were largely consistent across sociodemographic patient groups, with decision aids demonstrating similar efficacy when used with vulnerable patients such as the elderly and those with less income and less formal education [60]. These results are in conformity with the results of the current review, but evidence on the efficacy of decision aids in vulnerable groups is still scarce. If decision aids should be specifically tailored to such groups, for example by including appropriate visual illustrations (e.g., [61]) or animations (e.g., [56]) this could lead to an even better impact outcome in the decision making process. Although there are good overviews of available decision aids, such as The Ottawa Decision Aids Inventory gives, it is still unclear which formats are more or less applicable to older adults. A first follow up step could be to conduct a literature review on studies focusing on the design, application or evaluation of decision aids for older adults using other designs than RCTs and CCTs, including qualitative studies. This might help to identify existing decision aids for older adults and might give more insight whether there are more decision aids available for this group than those evaluated in RCTs and CCTs and whether these are designed age-appropriately. Moreover, it is recommended to tackle implementation strategies for decision aids to be used by older adults. There are already very relevant and useful Implementation Toolkits available (e.g., on the website https://decisionaid.ohri.ca/). However, it is still unclear whether interventions to improve adoption of SDM by health care providers are effective given the low quality of the evidence until now [62]. This certainly implies to implementation strategies among older people. Future research is needed to investigate which strategies work best in an older population.

A limitation of the current review is that we included studies using a sample with a *mean* age of 65 or older or reporting effectiveness analysis of the decision aid in a subsample of participants aged 65+ years. This means that part of the participants was younger than 65. Eleven of the nineteen studies used a cut-off point of at least 50 years to be eligible for the study. The other eight studies used no cut-off point. Although using a mean age may be misleading (e.g., if not normally distributed) we did not choose for a cut-off point as inclusion criterion. Our main aim was to investigate whether decision aids are effective in an (on average) older population. Given the lack of studies with a primary focus on the elderly, we also wanted to include studies that were conducted in an on average older population without a conscious focus on the elderly. In fact, the decision aids under investigation in the eight studies not using a cut-off point were aimed at improving decision making in treatment, screening or care decisions regarding diseases that are very common among older people, such as type II diabetes mellitus [33, 36, 48], atrial fibrillation [31, 35, 38], dementia [42] and prostate cancer screening [28]. In our opinion the results of these studies contributed to fulfilling the study goals.

Another limitation of this review is that we were not able to analyze the underlying evidence, and whether or not this evidence was appropriate for older people, which was unclear in most studies. We know from literature that evidence for many interventions in the older population is scarce, especially those with functional impairments and multi-morbidity [63]. We therefore expect that most decision aids did not tailor the evidence to this group. It is doubtful whether it will ever be possible to provide relevant and evidence based information tailored to all possible combinations of comorbidities in a single decision aid. This indicates that decision aids for older adults might be most effective when combined with high quality patient-provider interaction during consultation, with personal tailoring of the decision aid to the individual persons’ context. Although less often studied, the findings of this review indicate that decision aids have the potential to be effective in improving older adults’ participation in decision making and might improve patient-provider communication. Furthermore, it was found that patient outcomes were better when participants received the decision aid from their clinician during the consultation than when it was delivered by the researcher before the consultation [33, 48]. We also know that the health care provider is still the most important source of information for most older patients [64], and although some studies indicate that older adults have a preference for less participation in shared decision making than younger ones [65–67], other research shows that the majority of people, including older adults, want to be involved in the decision making process [10]. This suggests that decision aids might be particularly useful for older adults when successfully integrated with interpersonal communication in the consultation. There were only two other studies, described in three papers, in which the decision aid was delivered during the consultation [34, 39, 44], one of them using a trained research GP, so not the patients’ own GP [34, 44]. It is therefore recommended to study the added value of decision aids, delivered by the provider during the consultation, on communication during consultation in future research.

Last, there are some methodological limitations. Several outcome measurements are not validated for older adults. Moreover, there is little guidance on how to best assess outcomes measures such as knowledge and risk perception in decision aid trials. Few trials explicitly describe how these measures were developed. Furthermore, although most studies showed no risk of bias regarding random sequence generation and providing complete outcome data, there was a high risk of bias in more than 80 % of the studies with regard to blinding of participants and personnel and selective reporting. This is in line with the Cochrane review on the effectiveness of decision aids [12]. It is recommended to further diminish the risk of bias in future RCTs, particularly with regard to reporting bias.

## Conclusions

To conclude, the results of the current review indicate that decision aids can be effective for older adults. The decision aids increased their knowledge and risk perception, decreased decisional conflict and seemed to enhance participation in decision making. Further improvements can be made by a structured development and evaluation of those interventions among more heterogeneous groups of older adults. In particular, individualizing of decision aids to the heterogeneity and functional impairments of the oldest-old with, for instance, multi-morbidity and limited health literacy forms a challenge.

## Additional files

Additional file 1: Search Strategy. (DOCX 18 kb) Additional file 2: Characteristics of included studies. (DOCX 41 kb) Additional file 3: Results Meta-Analysis. (DOCX 161 kb) Additional file 4: Fulfilment of IPDAS criteria. (DOCX 24 kb) Additional file 5: Risk of Bias. (DOCX 15 kb)

## Abbreviations

CCT: controlled clinical trial
CG: control group
DCS: Decisional Conflict Scale
IG: intervention group
IPDAS: International Patient Decision Aid Standards
RCT: randomized controlled trial
SDM: shared decision making
