# Supplementary material for: Decision aids to help older people make health decisions: a systematic review and meta-analysis
Source: BMC Med Inform Decis Mak. 2016 Apr 21;16:45. doi: 10.1186/s12911-016-0281-8 (PMC4839148; doi:10.1186/s12911-016-0281-8)
Supplement: Additional file 1: — Search Strategy. (DOCX 18 kb) [file 12911_2016_281_MOESM1_ESM.docx]

**Additional file 1: Search Strategy**

Search date: 07-02-2014

MEDLINE(OvidSP) (December 2009-Februari 2014),

| Line | Term | n Documents |
| --- | --- | --- |
| 1 | choice behavior/ | 22612 |
| 2 | decision making/ | 70033 |
| 3 | exp decision support techniques/ | 63564 |
| 4 | Educational Technology/ | 1140 |
| 5 | decision$.tw. | 220396 |
| 6 | (choic$ or preference$).tw. | 314891 |
| 7 | communication package.tw. | 12 |
| 8 | or/1-7 | 601597 |
| 9 | exp health education/ | 138537 |
| 10 | Health Knowledge, Attitudes, Practice/ | 75146 |
| 11 | informed consent.tw,hw. | 47303 |
| 12 | patient.tw,hw. | 1900536 |
| 13 | consumer.tw,hw. | 65046 |
| 14 | or/9-13 | 2079973 |
| 15 | 8 and 14 | 151695 |
| 16 | ((patient$ or consumer$) adj1 (decision$ or choice or preference or participation)).tw. | 9570 |
| 17 | ((women or men) adj1 (decision$ or choice or preference or participation)).tw. | 288 |
| 18 | (parent$ adj1 (decision$ or choice or preferenc$ or participat$)).tw. | 2298 |
| 19 | ((personal or interpersonal or individual) adj (decision$ or choice or preference$ or participat$)).tw. | 3379 |
| 20 | shared decision making.tw. | 2322 |
| 21 | decision aid$.tw. | 1454 |
| 22 | informed choice.tw. | 998 |
| 23 | or/16-22 | 19275 |
| 24 | 15 or 23 | 159088 |
| 25 | clinical trial.pt. | 508112 |
| 26 | randomized controlled trial.pt. | 395746 |
| 27 | random$.tw. | 749625 |
| 28 | (double adj blind$).tw. | 122496 |
| 29 | double-blind method/ | 133141 |
| 30 | or/25-29 | 1144420 |
| 31 | 24 and 30 | 19789 |
| 32 | limit 31 to ed=20091201-20140201 | 6352 |

Embase(OvidSP)( December 2009-Februari 2014)

| Line | Term | n Documents |
| --- | --- | --- |
| 1 | *decision making/ | 40441 |
| 2 | *decision theory/ | 388 |
| 3 | decision$.tw. | 262165 |
| 4 | *Educational Technology/ | 817 |
| 5 | or/1-4 | 285425 |
| 6 | exp *health behavior/ | 94351 |
| 7 | exp *Patient Attitude/ | 60110 |
| 8 | exp *health education/ | 89464 |
| 9 | informed consent.tw,sh. | 75013 |
| 10 | patient.tw,sh. | 2394799 |
| 11 | consumer.tw,sh. | 54489 |
| 12 | or/6-11 | 2676800 |
| 13 | 5 and 12 | 93441 |
| 14 | ((patient$ or consumer$) adj1 (decision$ or choice or preference or participation)).tw. | 12241 |
| 15 | ((women or men) adj1 (decision$ or choice or preference or participation)).tw. | 303 |
| 16 | (parent$ adj1 (decision$ or choice or preferenc$ or participat$)).tw. | 2620 |
| 17 | ((personal or interpersonal or individual) adj (decision$ or choice or preference$ or participat$)).tw. | 3922 |
| 18 | shared decision making.tw. | 2580 |
| 19 | decision aid$.tw. | 1712 |
| 20 | informed choice.tw. | 1124 |
| 21 | or/14-20 | 23329 |
| 22 | 13 or 21 | 109290 |
| 23 | *Controlled Study/ | 2708 |
| 24 | *Randomized Controlled Trial/ | 7656 |
| 25 | *Clinical Study/ | 3006 |
| 26 | *Clinical Trial/ | 16007 |
| 27 | *Major Clinical Study/ | 8 |
| 28 | *Prospective Study/ | 4086 |
| 29 | *Multicenter Study/ | 2467 |
| 30 | *Randomization/ | 1134 |
| 31 | *Double Blind Procedure/ | 923 |
| 32 | *Single Blind Procedure/ | 52 |
| 33 | *Crossover Procedure/ | 698 |
| 34 | Placebo.tw,sh. | 315337 |
| 35 | random$.tw. | 869652 |
| 36 | (double adj blind$).tw. | 142900 |
| 37 | or/23-36 | 1089163 |
| 38 | 22 and 37 | 12513 |
| 39 | limit 38 to dd=20091201-20140201 | 6434 |

PsycINFO(OvidSP)(December 2009-Februari 2014)

| Line | Term | n Documents |
| --- | --- | --- |
| 1 | decision$.tw. | 138365 |
| 2 | (choic$ or preference$).tw. | 163122 |
| 3 | exp decision making/ | 62884 |
| 4 | computer assisted instruction/ | 12614 |
| 5 | or/1-4 | 294259 |
| 6 | exp health education/ | 13809 |
| 7 | exp health personnel attitudes/ | 16585 |
| 8 | informed consent.tw,sh. | 6461 |
| 9 | patient.tw,hw. | 175012 |
| 10 | consumer.tw,hw. | 38043 |
| 11 | exp health behavior/ | 16786 |
| 12 | or/6-11 | 256043 |
| 13 | 5 and 12 | 32025 |
| 14 | ((patient$ or consumer$) adj1 (decision$ or choice or preference or participation)).tw. | 4418 |
| 15 | ((women or men) adj1 (decision$ or choice or preference or participation)).tw. | 218 |
| 16 | (parent$ adj1 (decision$ or choice or preferenc$ or participat$)).tw. | 2913 |
| 17 | ((personal or interpersonal or individual) adj (decision$ or choice or preference$ or participat$)).tw. | 4136 |
| 18 | shared decision making.tw. | 1080 |
| 19 | decision aid$.tw. | 824 |
| 20 | informed choice.tw. | 342 |
| 21 | or/14-20 | 13463 |
| 22 | 13 or 21 | 40784 |
| 23 | random$.tw. | 125692 |
| 24 | (double adj blind$).tw. | 17902 |
| 25 | placebo$.tw,hw. | 30418 |
| 26 | or/23-25 | 146586 |
| 27 | 22 and 26 | 2764 |
| 28 | limit 27 to up=20091201-20140201 | 1121 |

Cochrane library central registry of studies (Whiley)(2010-2014)

| #1 | decision:ti,ab,kw |
| --- | --- |
| #2 | patient:ti,ab,kw |
| #3 | consumer:ti,ab,kw |
| #4 | #1 and (#2 or #3) |
| #5 | shared decision making:ti,ab,kw |
| #6 | decision aid*:ti,ab,kw |
| #7 | informed choice:ti,ab,kw |
| #8 | #4 or #5 or #6 or #7 from 2010 to 2014 |

Cinahl (EBSCO HOST) (1937-Febrauri 2014)

| S55 | S39 AND S52 |
| --- | --- |
| S54 | S39 AND S52 |
| S53 | S39 AND S52 |
| S52 | S40 OR S41 OR S42 OR S43 OR S44 OR S45 OR S46 OR S47 OR S48 OR S49 OR S50 OR S51 |
| S51 | AB ((singl* or doubl* or trebl* or tripl*) N1 (blind* or mask*)) |
| S50 | TI ((singl* or doubl* or trebl* or tripl*) N1 (blind* or mask*)) |
| S49 | (MH "Quantitative Studies") |
| S48 | AB placebo* |
| S47 | TI placebo* |
| S46 | (MH "Random Assignment") |
| S45 | AB random* |
| S44 | TI random* |
| S43 | AB (clinic* N1 trial*) |
| S42 | TI (clinic* N1 trial*) |
| S41 | PT clinical trial |
| S40 | (MH "Clinical Trials+") |
| S39 | S23 OR S38 |
| S38 | S23 OR S24 OR S25 OR S26 OR S27 OR S28 OR S29 OR S30 OR S31 OR S32 OR S33 OR S34 OR S35 OR S36 OR S37 |
| S37 | AB informed choice |
| S36 | TI informed choice |
| S35 | AB decision aid* |
| S34 | TI decision aid* |
| S33 | AB "shared decision making" |
| S32 | TI "shared decision making" |
| S31 | AB ((personal or interpersonal or individual) N1 (decision* or choice or preference* or participat*)) |
| S30 | TI ((personal or interpersonal or individual) N1 (decision* or choice or preference* or participat*)) |
| S29 | AB (parent$ N1 (decision* or choice or preferenc* or participat*)) |
| S28 | TI (parent$ N1 (decision* or choice or preferenc* or participat*)) |
| S27 | AB ((women or men) N1 (decision* or choice or preference or participation)) |
| S26 | TI ((women or men) N1 (decision* or choice or preference or participation)) |
| S25 | AB ((patient* or consumer*) N1 (decision* or choice or preference or participation)) |
| S24 | TI ((patient* or consumer*) N1 (decision* or choice or preference or participation)) |
| S23 | S9 AND S22 |
| S22 | S10 OR S11 OR S12 OR S13 OR S14 OR S15 OR S16 OR S17 OR S18 OR S19 OR S20 OR S21 |
| S21 | AB consumer |
| S20 | TI consumer |
| S19 | AB patient |
| S18 | TI patient |
| S17 | AB informed consent |
| S16 | TI informed consent |
| S15 | (MH "Consent+") |
| S14 | (MH "Professional Knowledge+") |
| S13 | (MH "Health Knowledge") |
| S12 | (MH "Health Education+") |
| S11 | (MH "Consumer Participation") |
| S10 | (MH "Health Behavior+") |
| S9 | S1 OR S2 OR S3 OR S4 OR S5 OR S6 OR S7 OR S8 |
| S8 | (MH "Educational Technology") |
| S7 | AB decision* |
| S6 | TI decision* |
| S5 | AB(choic* or preference*) |
| S4 | ti(choic* or preference*) |
| S3 | (MH "Help Seeking Behavior") |
| S2 | (MH "Information Seeking Behavior") |
| S1 | (MH "Decision Making+") |
