# Supplementary material for: Decision aids to help older people make health decisions: a systematic review and meta-analysis
Source: BMC Med Inform Decis Mak. 2016 Apr 21;16:45. doi: 10.1186/s12911-016-0281-8 (PMC4839148; doi:10.1186/s12911-016-0281-8)
Supplement: Additional file 2: — Characteristics of included studies. (DOCX 41 kb) [file 12911_2016_281_MOESM2_ESM.docx]

**Additional file 2: Characteristics of included studies (N=22)**

| **Reference** | **Methods** | **Participants** | **Age** | **Intervention group(s)** | **Control group(s)** | **Outcomes** |
| --- | --- | --- | --- | --- | --- | --- |
| Davison (1997) [28] | RCT | 60 participants; IG: 30; CG: 30  Diagnosis: prostate cancer  Gender: All male | Mean age: 67.9 (range 41-81).  EG: 66.0;  CG: 69.8 | Decision aid: written information package with discussion, a list of questions to ask their clinician, and an audiotape of the consultation, discussion with investigator and encouragement to read the information and use the list of questions to obtain the information needed to make a treatment decision. Delivered before initial treatment consultation with urologist. | Information package only: no encouragement from the researcher. | - Preferred role in treatment decision (before consultation) and assumed role in treatment decision (5-6 weeks after initial interview) - Anxiety - Depression |
| Dolan (2002) [29] | RCT | 95 participants; IG: 49; CG: 45  No diagnosis. The patients had an average risk of colorectal cancer  Gender (female):  IG: 53%; CG: 52% | Mean age: 66.2 (range 50-83).  IG: 65.0;  CG: 67.3  Inclusion criterium: 50 years or older. | Multicriteria-based decision aid for colorectal cancer screening, providing a conceptual representation of the decision and using it to organize and analyse information about expected outcomes, elicit values, and establish relationships between information and values.  IG also received a 2-part standardized interview prior to the doctor's appointment. The 1^st^ part was standardized and the 2nd part consisted of a detailed analysis of the decision regarding the recommended colorectal screening options using the analytic hierarchy process (AHP). Delivered before consultation with primary care physician. | The 1st part of the 2-part IG interview (consisting of a preliminary phase and an educational phase) prior to the doctor's appointment. | Process of decision making:   - Decisional conflict: feeling informed, clarity of values, ineffective decision making - Ratings of decisions they made (i.e. relationship between preferences and perceptions).   Decision outcomes:   - The proportion of colorectal cancer screening plans carried out. In cases where combined annual fecal occult blood tests and sigmoidscopy every 5 years was chosen, completion of either test by the time the chart was reviewed was counted as successful execution of this plan. |
| Fraenkel (2007) [30] | RCT | 83 participants. IG: 43; CG: 40  No diagnosis. Patients with self-reported knee pain on most days of the month.  Gender: not reported | Mean age: 74.  IG: 74; CG: 74.  Inclusion criterium: 60 years or older | Adaptive Conjoint Analysis (ACA) task (an interactive computer survey that enables patients to construct their treatment preferences by asking them to make tradeoffs between competing treatment characteristics in a series of rating tasks) and a handout illustrating their treatment preferences. The tasks elicited preferences for route of administration, benefits and side effects of commonly used options for knee pain. Delivered before consultation with primary care physician. | A general Arthritis Foundation (AFP) information pamphlet. | - Decisional self-efficacy (self-confidence in ability to participate in SDM) - Arthritis self-efficacy - Preparedness to participate (patients' perception of the usefulness of the intervention in preparing them to communicate with their physician) |
| Fraenkel (2012) [31] | RCT | 135 participants. IG: 69; CG: 66.  Diagnosis: NonValvular Atrial Fibrillation (NVAF)  Gender (female): IG: 1%; CG: 2%. | Age groups: 22.2% is < 65; 21.5% is 65-75; 56.3% is 75 years or older.  IG: <65: 25%; 65–74: 17%; 75–84: 46%; >85 12%.  CG: <65: 20%; 65–74: 26%; 75–84: 36%; >85 8%.  Decision aid was developed for older patients. | Decision aid: education about the connection between NVAF and stroke, about the different treatment options, and why treatment for NVAF involves a choice. The decision aid included individualized information regarding their risk of stroke and bleeding and the sequelae of these outcomes. Decision aid was completed before regularly scheduled visit. Delivered before consultation with primary care provider. | Usual care. | - Decisional conflict (subscales ‘Informed’ and ‘Values clarity’) - Knowledge - Accuracy of stroke risk and bleeding risk estimates - Patient-clinician communication - Anxiety - Worry about stroke - Worry about bleeding - Treatment preference - Change in treatment |
| Hanson (2011) [32] | RCT | 256 residents. IG: 127; CG: 129.  256 surrogates. IG: 127; CG: 129.  Diagnosis: Advanced dementia and feeding problems.  Gender (female residents): IG: 79%; CG: 76%.  Gender (female surrogates): IG: 68%; CG: 58%. | Mean age residents: 85.3.  IG: 85.2; CG: 85.3.  Mean age surrogates: 59.0.  IG: 59.3; CG: 58.7.  Inclusion criterium for residents: 65 years or older. | Audio or print decision aid on feeding options in advanced dementia. The decision aid provided information about dementia, feeding options and the outcomes, advantages, disadvantages of feeding tubes and assisted oral feeding, feeding for comfort near the end of life and discussed the surrogate’s role in decisions. Delivered during an enrollment interview. | Usual care. | Quality of decisional making   - Decisional conflict - Surrogate knowledge - Frequency of communication with providers / % feeding discussions - Feeling involved in feeding decisions - Satisfaction with decision making - Decisional regret   Treatments:   - Feeding treatment use |
| Jones (2009) [33]  See also  Weymiller (2007) | RCT | 98 participants; IG: 52; CG: 46  More specific: IG1: 26; IG2: 26; CG1: 23; CG2: 23  Diagnosis: Type 2 Diabetes Mellitus. Diagnosis of Coronary Artery Disease: IG: 50%; CG: 43%  Gender (female): IG: 31%; CG: 57%. More specific:  IG1: 26.9%; IG2: 34.6%;  CG1: 56.5%; CG2: 56.5% | Mean age: 65,4.  IG: 64; CG: 66.  More specific:  IG1: 65.4; IG2: 63.4; CG1: 67.4; CG2: 65.8 | Statin Choice decision aid, a single sheet designed for use during office visits that offered patients with diabetes probabilistic information, tailored to their estimated 10-year coronary heart disease risk, about the risks and benefits of using and not using statins. The decision aid also included a QPL. The intervention was delivered by either a researcher-diabetologist before the visit (IG1) or by the clinician during the office visit (IG2). | Mayo Clinic standard educational pamphlet about cholesterol management. The pamphlet defined lipid disorders and provided dietary guidelines for control of cholesterol along with general statements encouraging exercise and smoking cessation. No risk information and no QPL was included. The pamphlet was either delivered before the visit (CG1) or during the visit (CG2). | - Decisional conflict about statin use - Knowledge about statins and coronary risk |
| Kaner (2007) [34]  See also Thomson, 2007 | RCT | 29 participants: IG1: 11; IG2: 8; CG: 10.    Diagnosis: Atrial Fibrillation  Gender (female):  44,8%; no sign. differences in sex between groups. | Mean age: 72 (range 66-79).  No sign. differences in age between groups.  Inclusion criterium: 60 years or older. | Use of paper-based guidelines with two forms of computer-based decision aid. IG1: An implicit (concise) decision aid involved individualised risk and benefit presentation and a section to support SDM. IG2: An explicit (extended) decision aid additionally included patients' elicited values for health and treatment states derived via standard gamble and analysed in a Markov decision analysis. Next to personalised risk and benefit data, this version also derived personal values (utilities) for the relevant health states which were used in a decision analysis. The output of the decision analysis was presented to the patient to support the SDM section of the consultation. Delivered by trained research GPs who were not the patients’ usual GP during a referral consultation. | Paper-based guidelines only (without computer-based decision aid). | Communication duration:   - Median consultation time (duration opening activity, physical examination, presentation of risk information, decision-making discussions, closure work)   Communication content:   - Verbal behavior (verbal dominance, ratio technical vs. socio-emotional communication) - Non-verbal behavior (nodding, smiling, tool-directed gaze, head-shaking, pointing to patients) |
| Man-Son-Hing (1999) [35] | RCT | 263 participants; IG: 139; CG: 148  Diagnosis: Atrial Fibrillation  Gender (female): IG: 24%; CG: 24% | Mean age: 66.  IG: 65; CG: 67 | Decision aid: Audiobooklet explaining the results of a clinical trial and giving pertinent information to help patients decide whether to continue taking aspirin or switch to warfarin. It included audiotape, booklet and personal worksheet, existing of a value clarification section, QPL, preferred role in decision making and preferences for therapy. Delivered a few days before consultation with physician at Stroke Prevention in an Atrial Fibrillation (SPAF) center. | Usual care. | - Patient's ability to make choices regarding antithrombotic therapy - Knowledge - Expectations about the probability of stroke and major hemorrhage with aspirin or warfarin therapy - Decisional conflict - Satisfaction with the decision making process - Six-month adherence |
| Mathers (2012) [36] | RCT | 167 participants; IG: 89; CG: 78  Diagnosis: Type 2 Diabetes Mellitus  Gender (female): IG: 48%; CG: 43% | Mean age: 64.6 (range 39-87).  IG: 66; CG: 62 | Brief training of clinicians and use of PANDA decision aid on treatment choice in diabetes, including starting insulin, with patients in single consultation. Delivered before to consultation with GP or practice nurse. | Usual care. | Decision quality:   - Decisional conflict - Knowledge - Realistic expectations of the risks and benefits of insulin therapy and diabetic complications - Autonomy - Proportion undecided   Health outcome:   - Glycaemic control |
| Mathieu (2007) [37] | RCT | 734 participants; IG: 367; CG: 367  No diagnosis  Gender: all female | Mean age: 70.4. IG: 70.4; CG: 70.3  Inclusion criterium: 70 or 71 years | Paper booklet that consisted of information, a worksheet and an appendix. The information section described the options (to continue or stop screening) and the chances of each of the possible outcomes of each option. The worksheet contained a values clarification exercise. The appendix contained an explanation of the possibility of detecting a type of breast cancer that might not affect a woman’s health. The intervention was self-administered by the participants at home. | Usual care, i.e. standard Breast Screen NSW brochure containing a small amount of information regarding screening at different ages. It provided no numeric information about the outcomes of screening. | - Informed choice about whether to continue or stop screening (i.e. adequate knowledge and clear values and intention to either continue or stop mammography screening) - Proportion undecided - Decision (stop or continue screening) - Participating in screening. - Knowledge - Clarity of values - Decisional conflict - Attitudes towards screening - Anxiety - Breast cancer worry |
| McAlister (2005) [38] | RCT | 434 participants; IG: 219; CG: 215  Diagnosis: Atrial Fibrilation  Gender (female): IG: 43.4%; CG: 34.4% | Mean age: 72.  IG: 73; CG: 71. | Booklet and audiotape decision aid tailored to the patients’ personal stroke risk profile. The booklet described the potential consequences of NVAF-associated stroke or transient ischemic attack, provided patient-specific estimates of stroke risk and illustrated the potential benefits and risks of warfarin and ASA associated with each patient’s baseline risk. The intervention was self-administered by the participants at home. | Usual care. | - Change in the proportion of patients taking therapy appropriate to their stroke risk - Estimates of the potential benefits and risks of warfarin and ASA - Decisional conflict - Proportion of patients taking appropriate therapy at 12 months |
| Montori (2011) [39] | RCT | 100 participants; IG: 52; CG: 48  Diagnosis: Postmenopausal women with bone mineral density/t-scores less than -1.0 (consistent with a diagnosis of low bone mass/osteopenia or osteoporosis).  Gender (female): all female | Median age: 67.  IG: 67; CG: 67.  Inclusion criterium: 50 years or older. | Osteoporosis Choice decision aid providing the patient’s individualized 10-year risk estimate of having a major fracture (i.e., clinical spine, forearm, hip or shoulder fracture), calculated using data from the patient’s medical record, and describing the potential downsides of taking bisphosphonates. Clinicians were to discuss the decision aid during the consultation. Primary care clinicians delivered and discussed the decision aid during the consultation and patients took de decision aid home. | Usual care + the National Osteoporis Foundation booklet “Boning up on osteoporis: A guide to prevention and treatment”. | Survey:   - Knowledge - Satisfaction with knowledge transfer - Decisional conflict - Trust in physician   Video:   - Patient involvement in SDM   Medication adherence:   - Self-reported - Pharmacy records   Clinicians’ views:   - Perception of decision quality - Satisfaction with knowledge transfer |
| Partin (2004) [40]  See also Partin (2006) | RCT | 893 participants; IG1: 308; IG2: 295; CG: 290.  No diagnosis  Gender: all male | Mean age: 68.4.  IG1: 68,4; IG2: 68.4; CG: 68.3  Inclusion criterium: 50 years or older | 1. IG1) mailed video and IG2) mailed pamphlet about prostate cancer screening (PSA test). The interventions contained the same factual content. Video decision aid: message was conveyed by two providers describing their very different views about the value of the PSA test, followed by a message from a patient. The video was written at the 10th grade level, and sought to provide a balanced representation of the risks and benefits of screening. Pamphlet: conveyed in a more subtle approach, with the statement "not all doctors agree that PSA test should be done regularly" and explanation for why. The pamphlet, written at the 6th grade level was also designed to provide a balanced representation of the potential risks and benefits of screening. The point that there is a decision to make and that the patient should play an active role in it was emphasized throughout. Delivered by mail two weeks before primary care consultation. | Usual care. | - Knowledge - Participation (whether screening was discussed with provider) - PSA testing rates - Screening intention/ preferences (yes/no question regarding whether the patient thought they would have a PSA test in the next year) |
| Partin (2006) [41]  See also Partin (2004) | RCT | 893 participants; IG1: 308; IG2: 295; CG: 290.  No diagnosis  Gender: all male | Mean age: 68.4 (50-69: 52.4%; >70: 47.6%).  IG1: 68,4; IG2: 68.4; CG: 68.3  Inclusion criterium: 50 years or older | IG1) mailed video and IG2) mailed pamphlet. See Partin (2004) | Usual care. | - Knowledge |
| Stirling (2012) [42] | RCT | 31 participants; IG: 15; CG: 16.  Diagnosis: Healthy carers of people with Dementia  Gender (female): IG: 47.8%; CG: 52.2% | Mean age carers: 66.6 (range 42-90).  IG: 66.7; CG: 66.6 | Decision aid: Digital workbook mailed to carers of people with dementia. Participants were instructed to work through the contents of the decision aid over the following week. Both groups continued to receive usual care from community services. Delivered by mail. | Usual care (wait-listed). | - Carer burden - Decisional conflict - Knowledge of dementia |
| Street (1995) [43] | RCT | 60 participants  Diagnosis: Breast Cancer  Gender: All female | Mean age: 59.1 (range 35-82).  IG: 60.8; CG: 57.4  Comparison between older (> 65), less educated patients, and younger (< 65), more educated patients. | Multimedia program “Options for Treating Breast Cancer”: Interactive decision aid consisting of text, graphic display, audio narration, music, and audio-video clips. Several times throughout the program, the text and narration encourage the patient to ask questions, express concerns, and offer opinions when they visit with physicians. Delivered before the consultation with medical oncologist, radiation oncologist and surgeon. | Brochure “Care of Patients with Early Breast Cancer”. The medical information in the brochure was the same as that in the multimedia program, only in written form. The brochure also included several statements encouraging patients to ask questions, express concerns, and offer opinions. The brochure did not have a section comparable to the ‘Experiences of Other Women’ section of Options. | - Involvement in communication:   - Question-asking  - Opinion-giving  - Expression of concern   - Perceived decisional control - Perceived involvement in communication |
| Thomson (2007) [44]  See also Kaner, 2007 | RCT | 109 participants  IG: 53; CG: 56  Diagnosis: Atrial Fibrillation  Gender (female):  IG: 43.4%: 44.6% | Mean age: 73.4.  IG: 73.1;  CG: 73.7.  Inclusion criterium: 60 years or older. | Decision aid including individualized risk and benefit presentation and a section to support SDM. The individualised benefits/harms component included personalized risk assessment using the Framingham equation for stroke risk, and the benefits of Warfarin based on data of effectiveness from trials and the risks of bleeding based on a systematic review of literature. *Note:* the explicit arm (see Kaner (2007) was discontinued because participants found the elicitation of utilities to difficult. Delivered by trained research GPs who were not the patients’ usual GP during a referral consultation. | Direct doctor-led advice based on paper guidelines communicated directly to the participant by the clinic doctor. | - Decisional conflict - Anxiety - Knowledge - Treatment decision (starting or continuing Warfarin) - Use of primary and secondary care services - Health outcomes |
| Volandes (2009a) [45]  See also Volandes (2009b) and Volandes (2011) | RCT | 14 pairs: IG: 8 pairs; CG: 6 pairs.  Convenience sample of elderly patients not having moderate or severe cognitive impairment and surrogates visiting an urban geriatric clinic.  Gender (female):  Elderly patients: 50%  Surrogates: 78.6% | Mean age elderly patients: 83.  Mean age surrogates: 67.5  Inclusion criterium patients: 65 years or older | Participants were faced with the possibility of advanced dementia. decision aid: verbal narrative describing advanced dementia followed by viewing a 2-minute video decision aid visually depicting a patient with advanced dementia. The verbal narrative was read aloud by the interviewer and the video decision aid was viewed on a portable computer. Delivered by a trained member of the interview team in an urban geriatric clinic, followed by an interview. | Same verbal narrative describing advanced dementia (without video decision aid). | - Knowledge of advanced dementia - Concordance rate of preferences for goals of care (life prolonging care, limited care or comfort care) between patients and their surrogates. |
| Volandes (2009b) [46]  See also Volandes (2009a) and Volandes (2011) | RCT | 200 participants: IG: 94; CG: 106.  Convenience sample of elderly patients not having moderate or severe cognitive impairment visiting one of four clinics (urban geriatric clinic, suburban geriatric clinic, urban primary care clinic, suburban primary care clinic).  Gender (female): IG: 61%; CG: 56%. | Mean age: 75.  IG: 75; CG: 75.  Inclusion criterium patients: 65 years or older | Same as Volandes (2009a). Delivered by a trained member of the interview team in a primary care clinic or a geriatric clinic, followed by an interview. | Same as Volandes (2009a) | - Preferred goal of care in advanced dementia (life prolonging care, limited care or comfort care) - Preferences after six weeks |
| Volandes (2011) [47]  See also Volandes (2009a and 2009b) | RCT | 76 participants: IG: 33; CG: 43.  Convenience sample of elderly patients not having moderate or severe cognitive impairment visiting a rural primary care clinic.  Gender (female): IG: 58%; CG: 70%. | Mean age: 74.  IG: 73; CG: 75.  Inclusion criterium: 65 years or older. | Participants were faced with the possibility of advanced dementia. decision aid: verbal narrative describing advanced dementia followed by viewing a 6-minute video decision aid visually depicting a patient with advanced dementia. The verbal narrative was read aloud by the interviewer and the video decision aid was viewed on a portable computer. Narrative and first part of the video are the same as Volandes (2009a and 2009b), but video was extended with video images of the goals of care in advanced dementia, i.e. life-prolonging care images, limited medical care images and comfort care images. Delivered by a trained member of the interview team in a primary care clinic, followed by an interview. | Same as Volandes (2009a and 2009b) | Preferred goal of care in advanced dementia (life prolonging care, limited care or comfort care) |
| Weymiller (2007) [48]  See also Jones (2009) | RCT | See Jones (2009) | See Jones (2009) | See Jones (2009) | See Jones (2009) | - Acceptability - Knowledge about options and cardiovascular risk - Decisional conflict - Action (i.e., start of statin therapy) - Adherence to pill taking (statin use) after three months. |
| Wolf (2000) [49] | RCT | 399 participants: IG1: 130; IG2: 136: CG: 133.  Diagnosis: No diagnosis  Gender (female): IG1: 63%; IG2: 65%; CG: 62%. | Mean age: 74. IG1: 74; IG2: 74: CG: 75.  Inclusion criterium: 65 years or older. | Colorectal cancer screening informational script simulating an informed consent presentation about CRC screening, read aloud by a research assistant.  One of two informational interventions:  IG1: The relative risk reduction (RRR) script provided a 3-minute discussion of CRC screening methods, the mortality risk reduction described in terms of relative risk reduction (graphic provided) and the uncertain benefits of screening older persons;  IG2: The absolute risk reduction (ARR) was identical to the RRR information script, except that CRC mortality risk reduction was described in terms of absolute risk reduction (graphic provided). Delivered by a trained research assistant in primary care setting. | The control script briefly described CRC screening methods, i.e. fecal occult blood testing (FOBT) and flexible sigmoidoscopy. This information contained no stats and no graphics. | - Interest to begin or continue CRC screening, i.e. fecal occult blood testing (FOBT), flexible sigmoidoscopy or both. - Intent to begin or continue CRC screening, i.e. fecal occult blood testing (FOBT), flexible sigmoidoscopy or both. - Patients' estimate of FOBT positive predictive value for getting cancer (indicating comprehension) - Patients' estimates of colorectal cancer mortality reduction by screening (indicating perceived efficacy of screening) |

IG = intervention group; CG = control group; SDM = shared decision making
