# Supplementary material for: Decision aids to help older people make health decisions: a systematic review and meta-analysis
Source: BMC Med Inform Decis Mak. 2016 Apr 21;16:45. doi: 10.1186/s12911-016-0281-8 (PMC4839148; doi:10.1186/s12911-016-0281-8)
Supplement: Additional file 3: — Results Meta-Analysis. (DOCX 161 kb) [file 12911_2016_281_MOESM3_ESM.docx]

**Additional file 3: Results Meta-Analysis**

**Knowledge (dichotomous)**


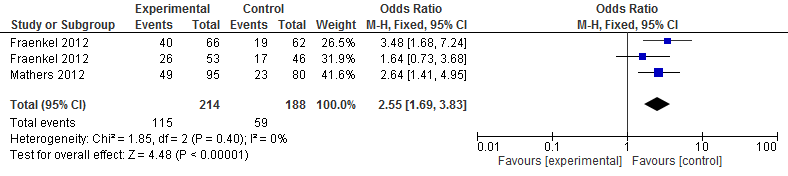


**Knowledge (continuous)**


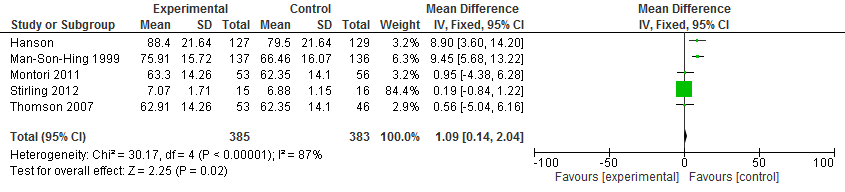


**Risk perception**


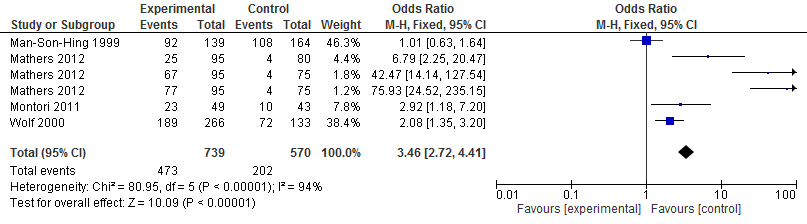


**DCS: Uncertainty**


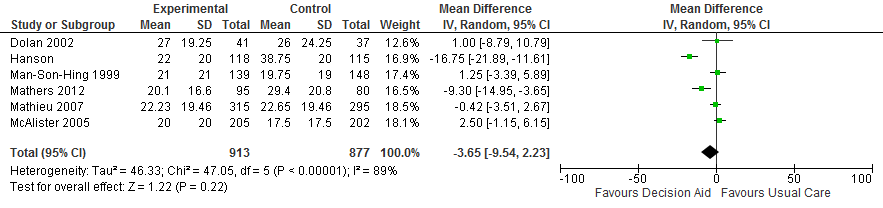


**DCS: Feeling uninformed**


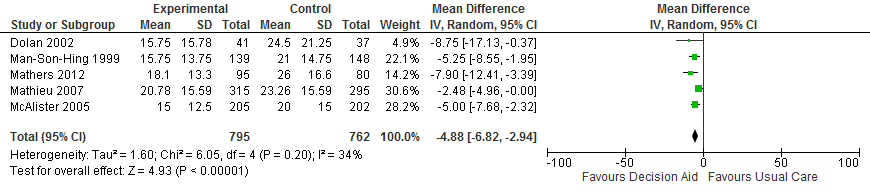


**DCS: Feeling unclear about values**


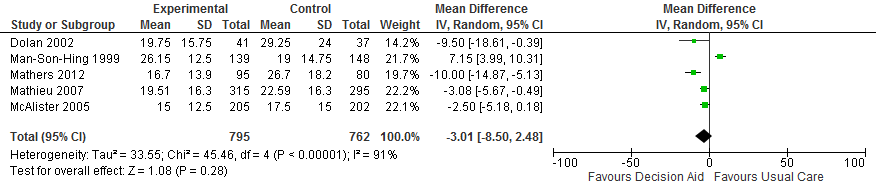


**DCS: Feeling unsupported**
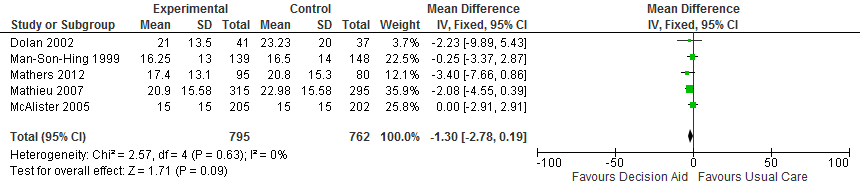


**DCS: Ineffective decision making**


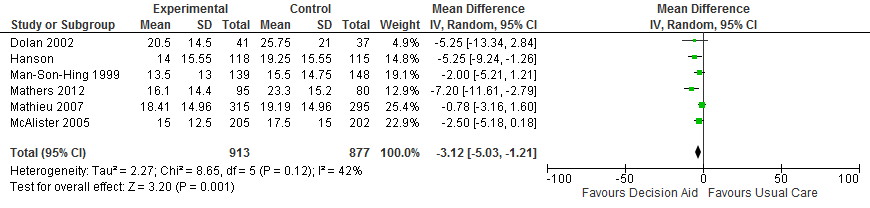


**DCS: Decisional conflict overall (total score)**


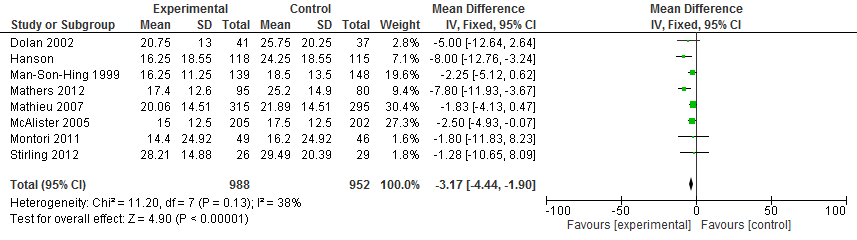


**Patient controlled decision making**


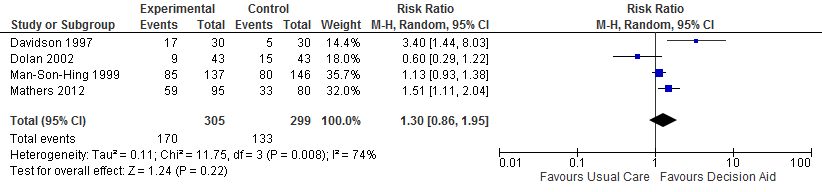


**Shared decision making**


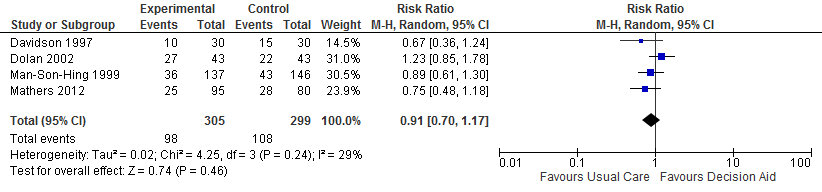


**Practitioner Controlled decision making**


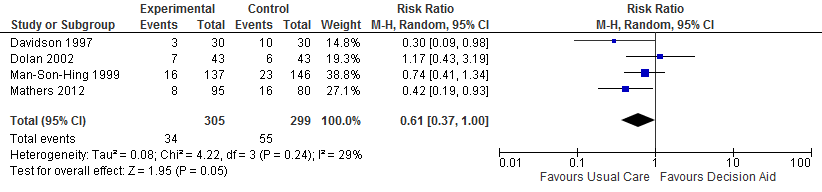


**Choice: Colorectal cancer screening**


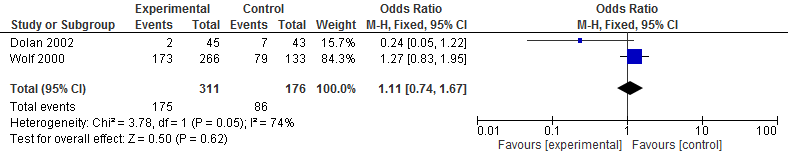


**Results of meta-analysis in Cochrane Review 2014 ^Ϯ^ (all ages) and current review (older adults)**

| **Outcome** | **Cochrane Review 2014^a^ (95% CI)** | **n** | **Current review**  **(95% CI)** | **n** |
| --- | --- | --- | --- | --- |
| Knowledge (dichotomous) | - | 0 | RR 1.71 (1.33, 2.18) | 2 |
| Knowledge (continous) | MD 13.34 (11.17, 15.51) | 42 | MD 6.50 (0.76, 12.25) | 5 |
| Risk perception | RR 1.82 (1.52, 2.16) | 19 | RR 2.27 (1.27, 4.06) | 3 |
| Decisional conflict (total) | MD -6.22 (-8.00, -4.44) | 28 | MD -3.17 (-4.44, -1.90) | 8 |
| 1. Uncertainty | MD -2.47 (-4.28, -0.66) | 23 | MD -3.65 (-9.54, 2.23) | 6 |
| 1. Uninformed | MD -7.26 (-9.73, -4.78) | 22 | MD -4.88 (-6.82, -2.94) | 6 |
| 1. Unclear values | MD -6.09 (-8.50, -3.76) | 18 | MD -3.01 (-8.50, 2.48) | 5 |
| 1. Unsupported | MD -4,77 (-6.86, -2.69) | 19 | MD -1.30 (-2.78, 0.19) | 5 |
| 1. Ineffective DM | MD -4.86 (-7.04, -2.68) | 19 | MD -3.12 (-5.03, -1.21) | 6 |
| Choice colorectal cancer screening | RR 1.12 (0.95, 1.31) | 10 | RR 0.67 (0.18, 2.54) | 2 |
| Patient Controlled Decision Making | RR 1.98 (1.02, 1.60) | 12 | RR 1.30 (0.86, 1.95) | 4 |
| Shared Decision Making | RR 0.96 (0.82, 1.13) | 12 | RR 0.91 (0.70, 1.17) | 4 |
| Practitioner Controlled Decision Making | RR 0.66 (0.53, 0.81) | 14 | RR 0.61 (0.37, 1.00) | 4 |

^a^ Stacey D, Légaré F, Col NF, Bennett CL, Barry MJ, Eden KB, Holmes-Rovner M, Llewellyn-Thomas H, Lyddiatt A, Thomson R, Trevena L, Wu JHC. **Decision aids for people facing health treatment or screening decisions.** *Cochrane Database Syst Rev* 2014, **1**: CD001431.
