# Supplementary material for: Decision aids to help older people make health decisions: a systematic review and meta-analysis
Source: BMC Med Inform Decis Mak. 2016 Apr 21;16:45. doi: 10.1186/s12911-016-0281-8 (PMC4839148; doi:10.1186/s12911-016-0281-8)
Supplement: Additional file 4: — Fulfilment of IPDAS criteria. (DOCX 24 kb) [file 12911_2016_281_MOESM4_ESM.docx]

**Additional file 4: Fulfilment of IPDAS criteria**

| **Short Title** | **1** | **2** | **3** | **4** | **5** | **6** | **7** | **8** | **9** | **10** | **11** | **12** | **13** | **14** | **15** | **16** | **17** | **18** | **19** | **25** | **26** | **27** | **28** | **Total** |
| --- | --- | --- | --- | --- | --- | --- | --- | --- | --- | --- | --- | --- | --- | --- | --- | --- | --- | --- | --- | --- | --- | --- | --- | --- |
| Davison (1997) | + | + | - | - | + | + | + | n/a | n/a | n/a | n/a | n/a | ? | n/a | n/a | n/a | + | ? | ? | ? | ? | ? | ? | **6/15 40.0%** |
| Dolan (2002) | + | + | + | **+** | **+** | **+** | **+** | **+** | **+** | **+** | **+** | **?** | **+** | **-** | **-** | **-** | **-** | **+** | **+** | **?** | **?** | **?** | **-** | **14/23 60.9%** |
| Fraenkel (2007) | + | + | + | **-** | **+** | **+** | **+** | **+** | n/a | n/a | n/a | n/a | **?** | **?** | **?** | **?** | **+** | **+** | **?** | **?** | **?** | **?** | **-** | **7/19 36.8%** |
| Fraenkel (2012) | + | + | + | **+** | **+** | **+** | **+** | **+** | n/a | n/a | n/a | n/a | **+** | **+** | **+** | **+** | **+** | **+** | **+** | **?** | **?** | **?** | **-** | **15/19 78.9%** |
| Hanson (2011) | + | + | + | **+** | **+** | **+** | **+** | **+** | n/a | n/a | n/a | n/a | n/a | n/a | n/a | n/a | **-** | **?** | **?** | **?** | **?** | **?** | **+** | **9/15 60.0%** |
| Jones (2009) | + | + | + | **+** | **+** | n/a | **+** | **+** | n/a | n/a | n/a | n/a | **+** | **+** | **+** | **+** | **+** | **+** | **+** | **-** | **+** | **+** | **-** | **16/18 88.9%** |
| Kaner (2007) | + | + | + | **+** | n/a | **+** | **+** | **+** | n/a | n/a | n/a | n/a | **+** | **+** | **+** | **+** | **+** | **+** | **+** | **?** | **?** | **?** | **?** | **14/18 77.8%** |
| Man-Son-Hing (1999) | + | + | + | **-** | **+** | **+** | **+** | **+** | **+** | n/a | n/a | n/a | **?** | **+** | **+** | **+** | **+** | **+** | **+** | **?** | **?** | **?** | **?** | **14/20 70.0%** |
| Mathers (2012) | + | + | + | **+** | **+** | **+** | **+** | **+** | n/a | n/a | n/a | n/a | **?** | **?** | **?** | **?** | **+** | **?** | **?** | **?** | **?** | **?** | **-** | **9/19 47.4%** |
| Mathieu (2007) | + | + | + | + | + | + | + | + | + | + | + | + | + | + | + | + | + | + | + | + | + | - | - | **21/23 91.3%** |
| McAlister (2005) | + | + | + | + | n/a | + | + | + | n/a | n/a | n/a | n/a | ? | ? | ? | n/a | + | ? | ? | ? | ? | ? | ? | **8/17 47.1%** |
| Montori (2011) | + | + | + | + | + | + | + | + | n/a | n/a | n/a | n/a | + | + | + | + | + | + | + | + | - | - | - | **16/19 84.2%** |
| Partin (2004) | + | + | + | + | + | + | + | ? | + | + | ? | ? | ? | ? | ? | ? | + | + | ? | ? | ? | ? | + | **12/23 52.2%** |
| Partin (2006) | + | + | + | + | + | + | + | ? | + | + | ? | ? | ? | ? | ? | ? | + | + | ? | ? | ? | ? | + | **12/23 52.2%** |
| Stirling (2012) | + | + | + | + | + | + | + | n/a | n/a | n/a | n/a | n/a | n/a | n/a | n/a | n/a | + | + | ? | ? | ? | ? | ? | **9/14 64.3%** |
| Street (1995) | + | + | + | - | + | - | - | - | n/a | n/a | n/a | n/a | ? | ? | ? | ? | + | ? | + | ? | - | - | n/a | **6/18 33.3%** |
| Thomson (2007) | + | + | + | + | n/a | + | + | + | n/a | n/a | n/a | n/a | + | + | + | + | + | + | + | ? | ? | ? | ? | **14/18 77.8%** |
| Volandes (2009a) | + | + | + | - | + | + | + | - | n/a | n/a | n/a | n/a | n/a | n/a | n/a | n/a | - | + | ? | - | - | - | n/a | **7/14 50.0%** |
| Volandes (2009b) | + | + | + | - | + | + | + | - | n/a | n/a | n/a | n/a | n/a | n/a | n/a | n/a | - | + | ? | - | - | - | n/a | **7/14 50.0%** |
| Volandes (2011) | + | + | + | - | + | + | + | - | n/a | n/a | n/a | n/a | n/a | n/a | n/a | n/a | - | + | ? | - | - | - | n/a | **7/14 50.0%** |
| Weymiller (2007) | + | + | + | + | n/a | + | + | + | n/a | n/a | n/a | n/a | + | + | + | + | + | + | + | - | + | + | - | **16/18 88.9%** |
| Wolf (2000) | + | + | + | + | + | + | + | + | + | + | + | - | - | + | ? | + | - | + | + | - | - | - | n/a | **15/22 68.2%** |
| **Total** | **22/22** | **22/22** | **21/22** | **15/ 22** | **17/ 17** | **20/ 22** | **20/22** | **14/ 20** | **6/6** | **5/5** | **3/5** | **1/5** | **8/18** | **9/16** | **8/16** | **9/15** | **15/ 22** | **17/ 22** | **11/ 22** | **2/22** | **3/22** | **1/22** | **3/17** |  |
| **Total %** | **100** | **100** | **95.5** | **68.2** | **100** | **90.9** | **90.9** | **70.0** | **100** | **100** | **60.0** | **20.0** | **44.4** | **56.3** | **50.0** | **60.0** | **68.2** | **77.3** | **50.0** | **90.9** | **13.6** | **4.5** | **17.6** |  |

n/a not applicable

DALI-IPDAS-1: The decision aid describes the condition (health or other) related to the decision.
DALI-IPDAS-2: The decision aid describes the decision that needs to be considered (the index decision).
DALI-IPDAS-3: Specific Options #1 to N
DALI-IPDAS-4: The decision aid describes what happens in the natural course of the condition (health or other) if no action is taken.

DALI-IPDAS-5: The decision aid has information about the procedures involved (e.g. what is done before, during, and after the health care option).
DALI-IPDAS-6: The decision aid has information about the positive features of the options (e.g. benefits, advantages).
DALI-IPDAS-7: The decision aid has information about negative features of the options (e.g. harms, side effects, disadvantages).

DALI-IPDAS-8: The information about outcomes of options (positive and negative) includes the chances they may happen.

DALI-IPDAS-9: If dealing with screening tests, the decision aid has information about what the test is designed to measure.

DALI-IPDAS-10: If dealing with screening tests, describes possible next steps based on the test results.

DALI-IPDAS-11: If dealing with screening tests, has information about the chances of disease being found with and without screening.

DALI-IPDAS-12: If dealing with screening tests, has information about detection and treatment of disease that would never have caused problems if screening had not been done.

DALI-IPDAS-13: If probabilities presented, are they stratified according to risk level. If quantitative, presentation. If numerical, the decision aid presents probabilities using event rates in a defined group of people for a specified time.

DALI-IPDAS-14: If numerical, do probabilities use the same denominator.

DALI-IPDAS-15: If numerical, do probabilities use the same time period.

DALI-IPDAS-16: If graphical, do probabilities use the same scale.
DALI-IPDAS-17: Values clarification included
DALI-IPDAS-18: The decision aid makes it possible to compare the positive and negative features of the available options.
DALI-IPDAS-19: Shows benefits and risks with equal detail (font, order display of statistics).

DALI-IPDAS-25: The decision aid provides references to scientific evidence used.
DALI-IPDAS-26: The decision aid reports the date when it was last updated.

DALI-IPDAS-27: The decision aid reports whether authors of the decision aid or their affiliations stand to gain or lose by choices people make after using the decision aid.

DALI-IPDAS-28: The decision aid (or available technical document) reports readability levels.
