# Supplementary material for: Decision aids to help older people make health decisions: a systematic review and meta-analysis
Source: BMC Med Inform Decis Mak. 2016 Apr 21;16:45. doi: 10.1186/s12911-016-0281-8 (PMC4839148; doi:10.1186/s12911-016-0281-8)
Supplement: Additional file 5: — Risk of Bias. (DOCX 15 kb) [file 12911_2016_281_MOESM5_ESM.docx]

**Additional file 5: Risk of Bias**

| Short Title | Random sequence generation | Allocation concealment | Blinding of participants and personal | Blinding of outcome assesment | Incomplete outcome data | Selective reporting | Other Bias |
| --- | --- | --- | --- | --- | --- | --- | --- |
| Davison (1997) | + | ? | ? | ? | + | ? | + |
| Dolan (2002) | + | + | ? | ? | + | ? | + |
| Fraenkel (2007) | + | ? | - | - | + | ? | - |
| Fraenkel (2012) | - | - | - | + | + | ? | + |
| Hanson (2011) | + | + | - | - | + | ? | + |
| Jones (2009) | + | + | - | + | ? | + | + |
| Kaner (2007) | ? | ? | - | - | - | ? | + |
| Man-Son-Hing (1999) | + | + | - | ? | + | ? | + |
| Mathers (2012) | + | - | - | - | + | ? | ? |
| Mathieu (2007) | + | + | - | + | + | ? | + |
| McAlister (2005) | + | + | - | + | + | ? | + |
| Montori (2011) | + | + | ? | + | + | + | + |
| Partin (2004) | + | ? | + | + | + | ? | + |
| Partin (2006) | + | ? | + | + | + | ? | + |
| Stirling (2012) | + | ? | - | - | + | ? | - |
| Street (1995) | ? | ? | ? | ? | ? | ? | + |
| Thomson (2007) | + | + | + | + | + | + | + |
| Volandes (2009a) | + | ? | ? | - | + | ? | + |
| Volandes (2009b) | + | ? | ? | - | + | ? | + |
| Volandes (2011) | + | ? | - | - | + | ? | + |
| Weymiller (2007) | + | + | + | + | + | ? | + |
| Wolf (2000) | ? | ? | ? | ? | ? | ? | + |
